# Advanced oxidation processes for the removal of antidepressants from wastewater: a comprehensive review

**DOI:** 10.1039/d5ra07764h

**Published:** 2025-12-08

**Authors:** Harez Rashid Ahmed, Anu Mary Ealias, Giphin George

**Affiliations:** a Department of Chemistry, College of Science, University of Sulaimani Qlyasan Street, Kurdistan Regional Government Sulaymaniyah 46001 Iraq harez.ahmed@univsul.edu.iq; b College of Science, Department of Medical Laboratory Science, Komar University of Science and Technology 46001 Sulaimani Iraq; c Department of Civil Engineering, VIT Mauritius, Uniciti International Education Hub 72448 Pierrefonds Mauritius; d Department of Mechanical Engineering, Koneru Lakshmaiah Education Foundation, Green Fields Vaddeswaram Andhra Pradesh-522302 India

## Abstract

Pharmaceutical contaminants, particularly antidepressants, have emerged as a critical environmental concern due to their persistence in aquatic ecosystems and potential toxicological effects. Despite partial removal through conventional wastewater treatment plants (WWTPs) and sewage treatment plants (STPs), residual concentrations ranging from nanograms to micrograms per liter persist, leading to adverse ecological consequences. Studies have demonstrated that even trace levels of selective serotonin reuptake inhibitors (SSRIs) and serotonin-norepinephrine reuptake inhibitors (SNRIs) can disrupt the physiological and behavioral processes of aquatic organisms, contributing to bioaccumulation and long-term ecological imbalances. Conventional techniques fail to achieve complete mineralization of antidepressants, necessitating the development of advanced remediation strategies. Advanced Oxidation Processes (AOPs) have emerged as a promising alternative, utilizing highly reactive species such as hydroxyl radicals (˙OH) and sulfate radicals (SO_4_˙^−^) to degrade complex pharmaceutical residues into harmless byproducts. This review systematically examines the sources, pathways, and environmental impact of antidepressants in water bodies while evaluating the efficiency and applicability of AOPs for their removal. A critical comparison of various AOPs, including photocatalysis, Fenton-like processes, ozonation, and sulfate radical-based oxidation, highlights their effectiveness in degrading antidepressants. The review discusses energy demand, byproduct formation, and cost-effectiveness, and proposes future perspectives for optimizing AOPs to enhance environmental sustainability.

## Abstract 2

Pharmaceutical contaminants, particularly antidepressants, have emerged as a critical environmental concern due to their persistence in aquatic ecosystems and potential toxicological effects.

## Graphical abstract

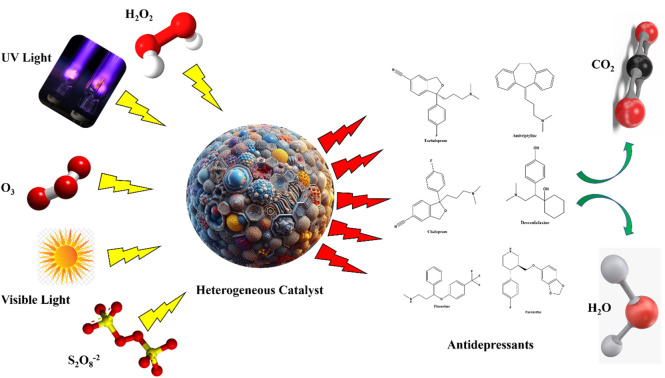

## 1 Introduction

Psychiatric disorders have become a significant global socioeconomic issue, with depression being one of the most prevalent mental health conditions. Among the most commonly prescribed treatments for depressive disorders are antidepressants (ATDs), which act on the central nervous system by modulating neurotransmitter concentrations such as serotonin, dopamine, and norepinephrine to elevate mood.^1^

As of 2019, these accounted for approximately 64.9%, 16.8%, and 5.7% of total ATD prescriptions, respectively.^1,2^ The increasing prevalence of psychiatric disorders, coupled with societal stressors, has led to a dramatic rise in ATD consumption worldwide. Between 2008 and 2018, global ATD prescriptions rose from 13.20 to 19.76 defined daily doses per 1000 inhabitants per day. However, due to incomplete metabolism in the human body, a significant portion of these pharmaceuticals is excreted as parent compounds or active metabolites *via* urine and feces. Consequently, the continuous release of ATDs into aquatic environments has resulted in their classification as “pseudo-persistent” contaminants, with reported surface water concentrations ranging from nanograms per liter (ng L^−1^) to micrograms per liter (µg L^−1^).^3^

Recent studies confirm the presence of these antidepressants in treated wastewater at detectable concentrations. For instance, venlafaxine levels up to 2.19 µg L^−1^ and duloxetine levels of 1.9 ng L^−1^ were found in secondary-treated effluents from the Metropolitan Wastewater Treatment Plant in St. Paul, Minnesota. Additionally, venlafaxine was detected at a concentration of 2.01 µg L^−1^ in treated sewage samples from Catalonia, Spain. In contrast, duloxetine, bupropion, and venlafaxine were detected at concentrations of 1.2, 50, and 900 ng L^−1^ in samples collected 1.7 km downstream from the Pecan Creek treatment plant in Texas.^4^

The primary pathways through which ATDs enter aquatic environments include excretion and improper disposal of unused medications. Due to their physicochemical properties, conventional wastewater treatment plants (WWTPs) struggle to remove pharmaceuticals effectively.^11,12^ Many pharmaceuticals are designed to be stable within the human body, making them resistant to degradation in wastewater treatment facilities and natural environments. Factors such as hydrophobicity, acidity, molecular structure, and polarity determine a compound's persistence and resistance to biodegradation.^11–16^ Consequently, ATDs remain in treated wastewater, raising concerns about their potential ecological and human health impacts.^5–7^

Although conventional treatment methods, such as activated sludge and membrane filtration, achieve partial ATD removal, their efficiency remains inadequate. Advanced oxidation processes (AOPs) have emerged as a promising solution due to their ability to generate highly reactive radical species, such as hydroxyl radicals (˙OH) and sulfate radicals (SO_4_˙^−^), which can effectively degrade ATDs.^8,9^ Recent research has explored the degradation of venlafaxine using advanced oxidation processes (AOPs), including UV/H_2_O_2_ and TiO_2_ photocatalysis, with the aim of identifying degradation pathways and assessing the toxicity of transformation products (TPs).^17–19^ Heterogeneous photocatalysis, particularly with TiO_2_ and ZnO, has demonstrated high efficiency in degrading organic pollutants due to its non-toxic, chemically inert, and cost-effective properties.^11,12^ Doping these materials with transition metals such as iron and cerium enhances photocatalytic activity by reducing charge recombination.^13–15^ Furthermore, (photo)-Fenton processes utilizing iron-based catalysts have shown promise for ATD degradation. Heterogeneous Fenton catalysts offer advantages over homogeneous systems by minimizing sludge formation and facilitating the recovery and reuse of the catalyst.^116^ Recent studies indicate that stabilizing Fe(ii) ions in magnetite structures using humic acid coatings (Fe_3_O_4_/HA) enhances Fenton-like activity, effectively activating H_2_O_2_ and persulfate for pollutant degradation.^17–22^ While Trawiński and Skibiński (2017) reviewed photodegradation of psychotropic drugs, this work extends beyond photolysis to analyze advanced oxidation processes (AOPs) such as Fenton-like, persulfate-based, and electrochemical oxidation. The present review also integrates data from 2020–2025, providing a broader mechanistic and sustainability perspective absent in the earlier work.^23^

Antidepressants exhibit strong chemical stability due to halogenated aromatic rings, tertiary amine groups, and electron-donating substituents, which hinder biodegradation and conventional treatment removal. This structural resilience highlights the need to understand their radical-mediated degradation mechanisms under AOP conditions. Despite the effectiveness of AOPs, challenges remain in fully elucidating pollutant degradation pathways and ensuring the complete mineralization of hazardous transformation products. Some pharmaceuticals, such as ibuprofen and naproxen, produce degradation by-products that exhibit significantly higher toxicity than their parent compounds.^24,25^ A comprehensive study of transformation products revealed that while most were less toxic or similarly toxic to their parent compounds, approximately 20% exhibited three times higher toxicity, and 9% were over ten times more toxic.^26–28^

Given these challenges, this review critically examines the application of Advanced Oxidation Processes (AOPs) in removing Advanced Treatment Discharge (ATD) from wastewater, focusing on three key AOPs and their respective mechanisms. The study aims to provide a comprehensive understanding of these processes' efficiency, limitations, and potential environmental implications, ultimately contributing to the development of sustainable wastewater treatment solutions.

## 2 Antidepressants as emerging contaminants

### 2.1 Types of antidepressants

While antidepressants may be the drug of choice for depression, they also have FDA approval as treatments for other medical disorders. For example, antidepressants help treat obsessive-compulsive disorder, social phobia, panic disorder, generalized anxiety disorder (GAD), and post-traumatic stress disorder (PTSD). Antidepressants also have non-FDA-approved, off-label indications. This activity reviews the indications, contraindications, actions, adverse events, and other key elements of antidepressant therapy in the clinical setting, as they relate to the essential points needed by members of an interprofessional team managing the care of patients receiving antidepressant medications for conditions that respond to this medication class.

While antidepressants are primarily indicated for the treatment of depression, they have received FDA approval for a range of other psychiatric and medical conditions. These include obsessive-compulsive disorder (OCD), social anxiety disorder, panic disorder, generalized anxiety disorder (GAD), and post-traumatic stress disorder (PTSD). In addition to their FDA approved indications, antidepressants are frequently prescribed for off-label uses, such as chronic pain syndromes, migraine prophylaxis, and sleep disorders. Given their broad therapeutic applications, a comprehensive understanding of their indications, contraindications, mechanisms of action, and potential adverse effects is essential for interprofessional teams managing patients who receive antidepressant therapy.

Depressive disorders encompass a spectrum of conditions, including unipolar major depressive disorder (MDD), persistent depressive disorder (dysthymia), premenstrual dysphoric disorder, and depression secondary to another medical condition. Among these, MDD is a highly disabling psychiatric disorder with significant morbidity and mortality. Epidemiological studies estimate the lifetime prevalence of MDD to range between 2% and 21% globally, with higher susceptibility observed in individuals of divorced marital status and females.^29^ Despite its debilitating impact, approximately 70% to 80% of individuals with MDD achieve symptom remission with appropriate pharmacological and psychotherapeutic interventions.

Antidepressants function by modulating key neurotransmitters implicated in mood regulation, including serotonin, norepinephrine, and dopamine. The major classes of antidepressants include.

#### 2.1.1 Selective serotonin reuptake inhibitors (SSRIs)

These are the most commonly prescribed antidepressants due to their favorable safety profile and efficacy. SSRIs selectively inhibit the serotonin transporter (SERT), increasing synaptic serotonin levels. Examples include fluoxetine, sertraline, and escitalopram.

#### 2.1.2 Serotonin-norepinephrine reuptake inhibitors (SNRIs)

These agents inhibit serotonin and norepinephrine reuptake, making them practical for mood and pain-related disorders. Common SNRIs include venlafaxine, duloxetine, and desvenlafaxine.

#### 2.1.3 Atypical antidepressants

This heterogeneous group includes agents that do not fit into conventional categories. Bupropion, for instance, acts as a norepinephrine-dopamine reuptake inhibitor (NDRI), whereas mirtazapine enhances noradrenergic and serotonergic transmission *via* α_2_-adrenergic antagonism.

#### 2.1.4 Serotonin modulators

These drugs exert dual action by inhibiting serotonin reuptake while directly modulating serotonin receptor subtypes. Trazodone and vilazodone are notable examples.

#### 2.1.5 Tricyclic antidepressants (TCAs)

Although historically a mainstay of depression treatment, TCAs are now prescribed less frequently due to their significant anticholinergic and cardiotoxic side effects. They inhibit the reuptake of serotonin and norepinephrine but interact with histaminergic, cholinergic, and adrenergic receptors, contributing to their broad side-effect profile. Examples include amitriptyline and nortriptyline.

#### 2.1.6 Monoamine oxidase inhibitors (MAOIs)

These are among the oldest classes of antidepressants. They function by irreversibly inhibiting monoamine oxidase, the enzyme responsible for breaking down serotonin, norepinephrine, and dopamine. Due to dietary restrictions and drug interactions, MAOIs (*e.g.*, phenelzine and tranylcypromine) are reserved for treatment-resistant depression.

**Fig. 1:**
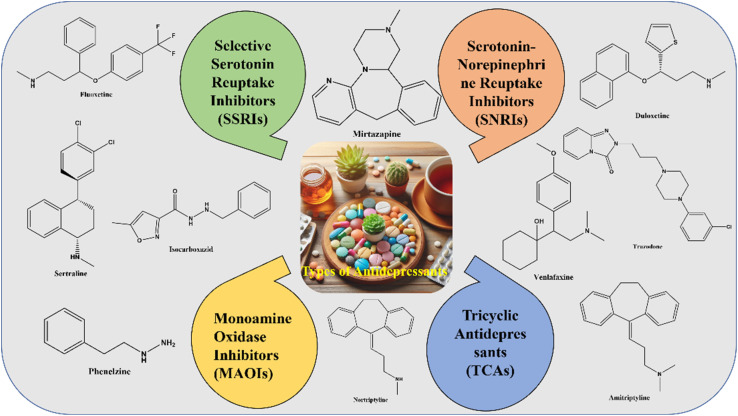
Classification of some antidepressants.

All currently approved antidepressants primarily enhance serotonergic, noradrenergic, or both neurotransmitter systems at the synapse. The precise mechanisms underlying their therapeutic effects remain under investigation; however, their efficacy is primarily attributed to sustained neurotransmitter elevation and receptor-level adaptations that occur over time.^30^ The pharmacodynamics and comparative properties of different antidepressant classes are summarized in Fig. 1 and Table 1.

**Table 1:** Some types of antidepressants

SSRIs	SNRIs	MAO-I	TCA	NARI	NaSSA
Citalopram	Venlafaxine	Tranylcypromine	Trimipramine	Viloxazine	Aptazapine
Escitalopram	Desvenlafaxine	Phenelzine	Amitriptyline	Tandamine	Esmirtazapine
Fluoxetine	Levomilnacipran	Selegiline	Imipramine	Talsupram	Mianserin
Paroxetine	Duloxetine	Isocarboxazid	Protriptyline	Amedalin	Mirtazapine
Sertraline			Nortriptyline	Atomoxetine	Setiptiline
Vortioxetine			Amoxapine	Daledalin	Teciptiline
Vilazodone			Desipramine	Edivoxetine	

### 2.2 Sources and pathways

**Fig. 2:**
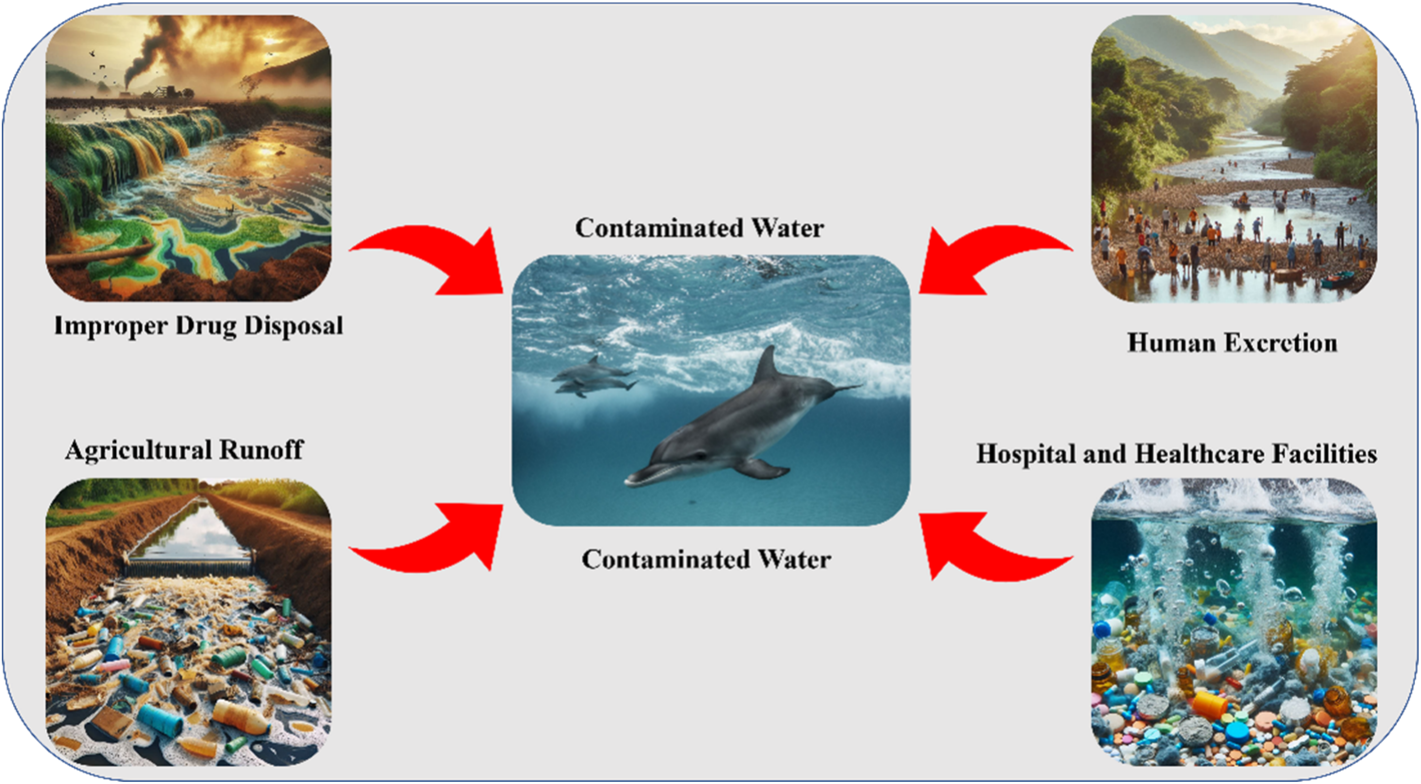
Pathways of antidepressant contamination in aquatic environments.

The presence of pharmaceutical residues, including antidepressants, antibiotics, β-blockers, non-steroidal anti-inflammatory drugs (NSAIDs), antiretroviral drugs, hormones, and lipid regulators, in aquatic environments has raised significant concerns due to their potential adverse effects on human health and ecological systems.^31^ Even at trace concentrations (ng L^−1^), these persistent contaminants can have severe consequences, including antimicrobial resistance, endocrine disruption, infertility, carcinogenesis, and reduced growth in plants and animals.^32^ Pharmaceutical pollutants primarily enter aquatic ecosystems through various anthropogenic activities. Key pathways include discharges from domestic and industrial sewage, leaching from landfills, improper disposal of domestic and hospital waste, and stormwater runoff.^33^ Aquatic environments serve as the ultimate sinks for these contaminants, with primary contributing sources including pharmaceutical manufacturing plants, domestic wastewater effluents, hospitals, veterinary clinics, agricultural runoff, and stormwater from farmland.^34^ Antidepressants, a widely detected class of pharmaceutical pollutants, enter aquatic systems through multiple pathways, as illustrated in Fig. 2:

Human excretion is the predominant source, as antidepressants and their active or inactive metabolites are eliminated *via* urine and feces. Conventional wastewater treatment plants (WWTPs) often lack the efficiency to fully degrade these compounds, resulting in their persistence in the treated effluent.

Improper drug disposal is flushing unused or expired medications down toilets or sinks, significantly contributing to water contamination.

Pharmaceutical manufacturing waste is effluent from drug production facilities and may contain high concentrations of antidepressant compounds, exacerbating contamination levels in receiving water bodies.

Hospitals and healthcare facilities introduce residues from antidepressants into wastewater streams through patient excretion and improper disposal of pharmaceutical waste in hospitals, psychiatric care centers, and other healthcare institutions.

Agricultural runoff is applying sewage sludge (biosolids) as fertilizers on agricultural land, which can introduce antidepressant residues into the soil and leach into surface and groundwater systems.

### 2.3 Environmental impact

Antidepressants have received increasing attention due to their significant environmental impact, particularly their risks to aquatic wildlife and their potential for misuse among consumers. Although wastewater treatment plants (WWTPs) and sewage treatment plants (STPs) can degrade these pharmaceuticals to some extent, residual concentrations ranging from nanograms to micrograms per liter persist in treated effluents, leading to adverse ecological effects. Several studies have demonstrated that even low concentrations of selective serotonin reuptake inhibitors (SSRIs) and serotonin-norepinephrine reuptake inhibitors (SNRIs) can disrupt critical biological processes in aquatic species.^35^

For instance, exposure to fluoxetine, as well as tricyclic antidepressants (TCAs) such as amitriptyline and mianserin, has been shown to alter gene transcription in zebrafish significantly (*Danio rerio*)^34^ Additionally, fluoxetine compromises the antipredator behavior of mosquitofish (*Gambusia holbrooki*), increasing their locomotor activity regardless of predator presence, which may reduce their survival rates in natural environments.^36^ Similarly, even low doses of fluoxetine have been found to affect the behavior of the freshwater invertebrate, *Gammarus pulex*, which plays a crucial role in aquatic food webs.^37^ Bioaccumulation of antidepressants has also been documented; citalopram, sertraline, and venlafaxine have been detected in the liver and brain tissues of rainbow trout (*Oncorhynchus mykiss*) exposed to municipal effluent from a Swedish STP. Furthermore, fish in the wild are continuously exposed to a mixture of antidepressants, raising concerns about the cumulative effects of these pharmaceuticals on aquatic organisms and ecosystems.

Given the persistence and bioaccumulative potential of antidepressants, effective and highly sensitive analytical methods are essential to detect and quantify their environmental concentrations. However, conventional wastewater treatment methods are often insufficient to obliterate these contaminants. In this regard, Advanced Oxidation Processes (AOPs) have emerged as promising solutions for the degradation of persistent pharmaceutical residues, including antidepressants. AOPs generate highly reactive species, such as hydroxyl radicals (˙OH) and sulfate radicals (SO_4_˙^−^), which can effectively break down complex organic pollutants into non-toxic byproducts. Compared to conventional treatment methods, AOPs offer higher degradation efficiencies and broader applicability, making them a viable approach for mitigating the environmental impact of pharmaceutical contaminants.

## 3 Comparative performance of AOPs in antidepressant degradation

Advanced Oxidation Processes (AOPs) constitute a class of chemical treatment methods to eliminate organic and inorganic contaminants from water and wastewater. The fundamental principle underlying AOPs is the generation of highly reactive hydroxyl radicals (˙OH), among the most potent oxidizing agents known. These radicals facilitate the degradation of a broad spectrum of pollutants through hydrogen abstraction, electron transfer, and radical addition. The generation of ˙OH can be achieved through various techniques, including ozonation, photocatalysis, Fenton's reaction, and electrochemical oxidation, each of which operates under distinct chemical conditions but ultimately seeks to produce ˙OH to decompose complex pollutants into more straightforward, less hazardous compounds. Advanced oxidation processes (AOPs) differ significantly in radical generation pathways, energy consumption, and pollutant degradation efficiency. Comparative evaluation is crucial to identify the most sustainable and scalable system for antidepressant removal from aquatic environments. The efficiency of AOPs largely depends on the oxidant type, activation route, catalyst design, and operating parameters such as pH, temperature, and irradiation source.^10,38^

AOPs present several advantages over conventional wastewater treatment techniques. One of the primary benefits is their high efficiency in degrading recalcitrant organic pollutants that exhibit resistance to traditional biological treatments. The ˙OH generated in AOPs possesses a high oxidation potential, enabling the transformation of complex organic molecules into more straightforward, less toxic compounds.^39^ Another significant advantage is the capacity of AOPs to completely mineralize contaminants, meaning that organic pollutants are fully oxidized to carbon dioxide, water, and inorganic ions, thereby preventing the accumulation of harmful residues.^40^ This attribute is particularly relevant for the removal of pharmaceuticals such as antidepressants, which are known for their persistence and toxicity in aquatic environments. Additionally, AOPs demonstrate considerable versatility as they can be applied to various pollutants. Integrating AOPs with other treatment methodologies further enhances their efficiency; for example, combining AOPs with biological treatments can improve the biodegradability of wastewater, rendering it more amenable to conventional treatment processes.^41^ Furthermore, AOPs obviate the need for hazardous chemical additives for disinfection, reducing the risk of secondary pollution. The by-products formed during AOP treatments are generally less toxic than those generated by conventional processes.^40^

Recent research has substantiated the efficacy of AOPs in removing various pharmaceutical compounds, including antidepressants, from wastewater. For instance, Deng and Zhao^10^ reported the successful application of AOPs for treating landfill leachate and biologically treated municipal wastewater, demonstrating substantial removal of refractory organic pollutants. Similarly, Jaimes-López *et al.*^42^ investigated the role of heterogeneous catalysts in enhancing ˙OH generation within AOPs, highlighting the critical influence of catalyst performance and stability on process efficiency. Several studies have specifically examined the removal of antidepressants *via* AOPs. For example, Al Mayyahi and Al-Asadi.^43^ Reviewed the implementation of AOPs for eliminating pharmaceuticals from wastewater, reporting high removal efficiencies for compounds such as fluoxetine and sertraline. Among hydroxyl-based AOPs, photocatalytic oxidation using TiO_2_, ZnO, and g-C_3_N_4_ materials remains one of the most studied systems. These catalysts effectively generate ˙OH radicals under UV or visible light through electron–hole separation. Recent studies have shown that doped and composite catalysts—such as Fe–TiO_2_, Ag/ZnO, and BiVO_4_-graphene enhance light absorption and charge carrier mobility, achieving degradation efficiencies of over 90% for fluoxetine and venlafaxine after 60–120 min of irradiation. However, photocatalytic systems generally suffer from low solar utilization efficiency and catalyst fouling, which can limit large-scale implementation. These findings underscore the potential of AOPs as a robust and adaptable technology for treating wastewater containing persistent organic pollutants, including pharmaceutical residues, as illustrated in Table 2, a summary of various AOPs.

**Table 2:** Comparative performance of advanced oxidation processes (AOPs) for antidepressant degradation: oxidant generation, efficiency, and scalability

AOP method	Primary oxidant	Typical catalyst/System	Target pollutants	Removal efficiency (%)	Key advantages	Limitation	References
Ozonation	O_3_		Pharmaceuticals	80–95	No sludge formation		44
Photocatalysis	˙OH	TiO_2_, ZnO, g-C_3_N_4_	Fluoxetine, venlafaxine	85–95	Simple design, solar-driven	Low solar efficiency, fouling	45–50
Fenton/Photo-Fenton	˙OH	Fe^2+^/H_2_O_2_, Fe_3_O_4_ composites	Sertraline, amitriptyline	90–100	High oxidation power	Sludge formation, acidic pH	51–54
Persulfate (PMS/PDS)	SO_4_˙^−^/˙OH	Co^2+^, Fe^3+^, MnO_2_, carbon	Fluoxetine, citalopram	90–98	Neutral pH, strong radicals	Metal leaching, sulfate ions	55–57
Electrochemical/EF/PEF	˙OH, H_2_O_2_	BDD, SnO_2_, PbO_2_	Fluoxetine, venlafaxine	92–99	Highly efficient, tunable	High energy cost	58 and 59

### 3.1 Types of AOPs

#### 3.1.1 Fenton and photo-fenton processes

Rely on *in situ* formation of ˙OH radicals through the reaction between Fe^2+^ and H_2_O_2_. These systems are simple, inexpensive, and highly reactive, often achieving complete removal of antidepressants such as sertraline or amitriptyline within 60 min under acidic conditions (pH ≈ 3). Nevertheless, their dependency on acidic media and the production of iron-containing sludge represents notable drawbacks. Heterogeneous Fenton-like systems, such as Fe_3_O_4_/graphene or CuFe_2_O_4_ catalysts, have been developed to overcome these issues by improving reusability and reducing secondary pollution. Recent developments in photocatalytic materials have extended beyond traditional TiO_2_ systems to include a range of visible-light-responsive and magnetically separable photocatalysts. Graphene-supported Fe_3_O_4_ and CuFe_2_O_4_ composites have demonstrated dual functionality, combining photocatalytic and Fenton-like oxidation pathways that enhance reusability while minimizing secondary pollution. Similarly, graphitic carbon nitride (g-C_3_N_4_) and its heterojunctions with BiVO_4_, ZnO, or Ag_3_PO_4_ exhibit strong visible-light absorption, improved electron–hole separation, and superior degradation efficiency for antidepressants such as fluoxetine, venlafaxine, and sertraline. The synergy between semiconductor band alignment and reactive oxygen species (ROS) generation (˙OH, ˙O_2_^−^, and SO_4_˙^−^) has been reported to accelerate degradation kinetics and enhance mineralization efficiency significantly. These findings demonstrate that the rational design of multi-component photocatalysts can overcome TiO_2_'s limitations in narrow band gap and recombination losses, thereby offering promising routes for scalable and energy-efficient AOP applications.^60–63^

**Fig. 3:**
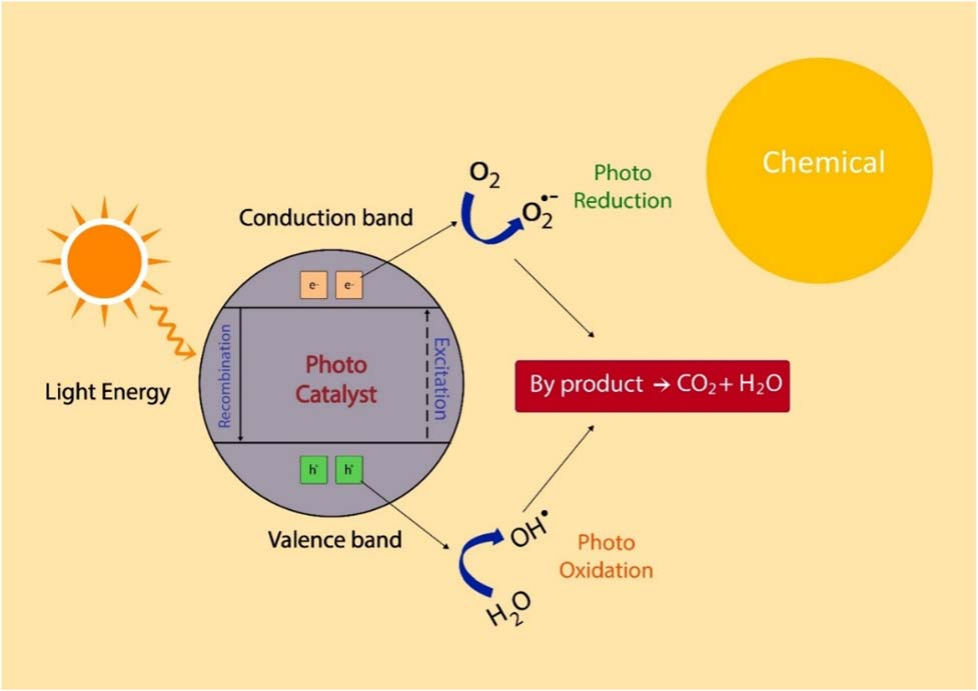
Demonstrates photocatalytic degradation mechanisms using TiO_2_-based catalysts, reproduced from ref. 65, Springer Nature Switzerland AG, Copyright © 2024.

Photocatalysis is a widely utilized AOP that employs light energy to activate a photocatalyst, typically titanium dioxide (TiO_2_), to generate reactive species capable of degrading organic pollutants Fig. 3. Upon exposure to ultraviolet (UV) or visible light, TiO_2_ absorbs photons, leading to the excitation of electrons from the valence band to the conduction band, thereby generating electron–hole pairs. These charge carriers interact with water and oxygen molecules to form highly reactive hydroxyl radicals (˙OH) and other reactive oxygen species (ROS), which are highly efficient in decomposing complex organic contaminants into simpler, non-toxic compounds.^64,65^

TiO_2_ remains the most widely employed photocatalyst due to its strong oxidative potential, chemical stability, non-toxicity, and cost-effectiveness. However, its relatively wide bandgap (3.2 eV for anatase) restricts its absorption primarily to the UV region, constituting only a tiny fraction of the solar spectrum.^66^ Several modifications have been implemented to enhance its visible light activity to overcome this limitation. These include non-metal doping (*e.g.*, nitrogen, carbon, and sulfur), metal doping (*e.g.*, silver, iron), and coupling with other semiconductors.^67^ Doping TiO_2_ with non-metals such as nitrogen (N), carbon (C), and sulfur (S) reduces its bandgap energy, enabling enhanced absorption in the visible spectrum. For example, nitrogen-doped TiO_2_ introduces mid-gap states that facilitate electron excitation, thereby improving photocatalytic activity under visible light.^68^ Metal doping, particularly with silver (Ag) and iron (Fe), enhances photocatalytic efficiency by suppressing electron–hole recombination, thereby prolonging charge carrier lifetimes and increasing the degradation rates of pollutants.^69^ Additionally, coupling TiO_2_ with semiconductors such as cadmium sulfide (CdS) and zinc oxide (ZnO) enables the formation of heterojunction structures that improve charge separation and expand the light absorption range. For instance, TiO_2_/CdS composites have demonstrated superior photocatalytic performance under visible light due to the synergistic interaction between both materials.^70,71^

Recent research has focused on optimizing the efficiency and applicability of TiO_2_ and alternative photocatalysts for environmental remediation. Mittal *et al.*^72^ conducted a comprehensive review on the impact of non-metal doping on the physicochemical properties and photocatalytic activity of TiO_2_, highlighting significant enhancements in visible light absorption and pollutant degradation efficiency. Similarly, Chauke *et al.*^67^ Explored the potential of TiO_2_-based photosensitizers for the photocatalytic degradation of thiazine dyes, demonstrating the promising role of dye-sensitized TiO_2_ in wastewater treatment applications. Furthermore, a study conducted by the Kennedy Space Center successfully developed an Ag-doped TiO_2_ catalyst with enhanced photocatalytic efficiency under visible light conditions, reinforcing the feasibility of modified TiO_2_ catalysts for real-world pollutant removal applications.^66^

Photocatalysis, particularly utilizing TiO_2_ and its modified derivatives, represents a highly effective and versatile AOP for wastewater treatment. Advances in doping strategies, semiconductor coupling, and sensitization techniques have significantly improved its photocatalytic efficiency and broadened its applicability under visible light irradiation. Continued research and innovation in this domain will further refine the practical deployment of photocatalysis for environmental remediation.

#### 3.1.2 Fenton process

The Fenton process is a widely studied AOP that employs the reaction between ferrous iron (Fe^2+^) and hydrogen peroxide (H_2_O_2_) to generate hydroxyl radicals (˙OH), which are highly reactive and effective in degrading organic pollutants in aqueous environments.^73,74^ The primary reactions in the Fenton process are as follows (eqn (1) and (2)):

1 Fe^2+^ + H_2_O_2_ → Fe^3+^ + OH˙ + OH^−^

2
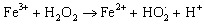


The ˙OH radicals generated in these reactions can attack and break down organic molecules into more straightforward, less harmful compounds.^75^

The Fenton process is highly efficient in degrading recalcitrant organic pollutants resistant to conventional biological treatments.^18–20,76^ The ˙OH radicals generated have a high oxidation potential, enabling the breakdown of complex molecules into more straightforward, less harmful compounds.^77^ One of the significant advantages of the Fenton process is its operational simplicity. It does not require sophisticated equipment or high-energy inputs, making it a cost-effective option for wastewater treatment.^78^ The process can be easily implemented in existing treatment facilities with minimal modifications. The Fenton process is versatile and can be applied to various pollutants, including pharmaceuticals, dyes, and industrial chemicals. It can also be integrated with other treatment processes to enhance overall efficiency. For example, combining the Fenton process with biological treatments can improve the biodegradability of wastewater, making it easier to treat with conventional methods.

Recent studies have focused on improving the efficiency and applicability of the Fenton process for environmental remediation. For instance, a survey by Kremer investigated the kinetics of modified versions of the Fenton reaction, highlighting the role of FeO^2+^ as an intermediate in the reaction mechanism.^75^ Another study reviewed the use of Fenton reaction systems for water treatment, emphasizing the importance of optimizing pH and other operational parameters to enhance process efficiency.^79^ Several case studies have demonstrated the practical applications of the Fenton process in wastewater treatment. For example, a survey by Deb *et al.* explored the use of the Fenton process to remove micro-pollutants from industrial wastewater, achieving significant reductions in pollutant concentrations.^80^ Another study reviewed the application of the Fenton process in wastewater treatment, highlighting its effectiveness in improving water quality and ensuring compliance with environmental regulations.^81^

With its Fe^2+^/H_2_O_2_ system, the Fenton process for ˙OH generation represents a powerful and versatile AOP for degrading organic pollutants in wastewater. Its high efficiency, operational simplicity, and versatility make it a superior alternative to conventional treatment methods. Continued research and development in this field will further enhance the applicability and effectiveness of the Fenton process in environmental remediation.

#### 3.1.3 Ozonation

Ozonation is an AOP that utilizes ozone (O_3_) as a strong oxidant to degrade organic pollutants in water and wastewater. O_3_ is a triatomic molecule known for its high oxidation potential. When dissolved in water, O_3_ decomposes to form hydroxyl radicals (˙OH), which are highly reactive and capable of breaking down complex organic molecules into more straightforward, less harmful compounds.^65,82^ The primary reaction involved in ozonation is given by (eqn (3)):

3 O_3_ + H_2_O → OH˙ + O_2_ + OH^−^

Both ˙OH and O_3_ contribute to the oxidation and mineralization of pollutants, making ozonation an effective method for wastewater treatment.^83^

Ran *et al.* explored ultrasound-assisted catalytic ozonation as a method for removing 5-hydroxy-1,3-phthalic acid from strongly alkaline and high-salt solutions, thereby contributing to nuclear waste disposal and human health.^84^ O_3_ has a higher oxidation potential than chlorine and H_2_O_2_, enabling it to degrade a wide range of organic pollutants, including pharmaceuticals such as antidepressants.^85^ Unlike conventional treatments, ozonation does not produce harmful by-products, as O_3_ decomposes into oxygen post-oxidation, making it an environmentally friendly option studied by Kaswan and Kaur.^82^ Sutar and Mane^26^ have demonstrated the effectiveness of ozonation in treating industrial wastewater and removing persistent organic pollutants, reinforcing its role as a powerful AOP for environmental remediation.^85^ Another study reviewed the application of ozonation in municipal wastewater treatment, highlighting its effectiveness in improving water quality and compliance with environmental regulations.^86^ Additionally, Ran *et al.* investigated the combined use of sonolysis and ozonation (US/O_3_) for removing organic compounds from Bayer liquor, achieving a total organic carbon (TOC) removal of 60.13% and a decolorization of 87.1%.^87^ Moreover, Ran *et al.* studied ultrasonic-induced crystallization for efficient extraction of hazardous sodium oxalate with ultra-low alkali loss in the alumina industry, demonstrating scalability from laboratory to continuous pilot scale.^88^

Ozonation, utilizing O_3_ as a strong oxidant, represents a powerful and versatile advanced oxidation process (AOP) for degrading organic pollutants in wastewater. Its high oxidation potential, broad spectrum of activity, and environmental benefits make it a superior alternative to conventional treatment methods. Continued research and development in this field will further enhance the applicability and effectiveness of ozonation in environmental remediation. Ran *et al.* examined the oxidation of 4-hydroxybenzoic acid in strongly alkaline and high-salt solutions using ultrasonic-assisted ozone, which aids in the disposal of radioactive waste and promotes environmental safety.^89^

#### 3.1.4 UV/H_2_O_2_ oxidation technology

The UV/H_2_O_2_ process is an advanced oxidation process (AOP) that combines ultraviolet (UV) light with hydrogen peroxide (H_2_O_2_) to generate hydroxyl radicals (˙OH), which are highly reactive and capable of degrading a wide range of organic pollutants in water and wastewater. The primary reaction in this process involves the photolysis of H_2_O_2_ under UV irradiation, leading to the formation of ˙OH (eqn (4)):

4 H_2_O_2_ + UV → 2OH˙

The ˙OH radicals produced in this reaction effectively break down complex organic molecules into more straightforward, less harmful compounds. This makes the UV/H_2_O_2_ process particularly efficient for treating recalcitrant pollutants, including pharmaceuticals such as antidepressants.

One of the key advantages of this process is its high oxidation potential, as ˙OH is among the most potent known oxidants. This enables the degradation of a broad spectrum of organic pollutants that are resistant to conventional treatment methods.^90^ The process has demonstrated high removal efficiencies for pharmaceuticals, pesticides, and industrial chemicals. Additionally, the operational simplicity of the UV/H_2_O_2_ system, requiring only UV lamps and H_2_O_2_ dosing, makes it relatively easy to implement and maintain.^91^

A significant benefit of the UV/H_2_O_2_ process is its environmental safety. Unlike other oxidation techniques, H_2_O_2_ decomposes into water and oxygen, leaving no harmful residues. Furthermore, the reaction products of pollutant degradation primarily include water, carbon dioxide, and inorganic ions, which pose minimal environmental risks.^92^ The process can also be integrated with complementary treatment methods, such as biological filtration or adsorption, to enhance overall treatment efficiency and pollutant removal.^93^

Recent research has focused on optimizing the efficiency of the UV/H_2_O_2_ process for environmental remediation. For example, Buthiyappan *et al.* reviewed the degradation performance and economic aspects of UV-based AOPs, including UV/H_2_O_2_, emphasizing optimizing parameters such as pH, oxidant concentration, and UV intensity to maximize process efficiency.^90^ Case studies further demonstrate its effectiveness in degrading specific contaminants. Afzal *et al.* (2012) investigated the decomposition of cyclohexanoic acid under various conditions using UV/H_2_O_2_, reporting significant removal efficiencies of.^94^ Similarly, AlHamedi *et al.* (2009) studied the degradation of Rhodamine B under UV/H_2_O_2_ treatment, highlighting the process's capability to break down complex organic pollutants.^95^

Overall, the UV/H_2_O_2_ process represents a highly effective and versatile AOP for wastewater treatment. Its strong oxidation capacity, ease of operation, and environmental compatibility make it a promising alternative to conventional treatment methods. Ongoing advancements in process optimization will further enhance its applicability in environmental remediation.

#### 3.1.5 Persulfate-based AOPs (PS-AOPs)

**Fig. 4:**
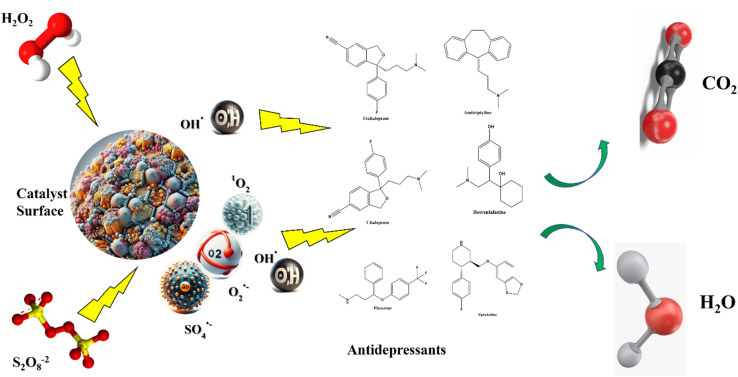
Proposed degradation pathways of fluoxetine under hydroxyl (˙OH) and sulfate (SO_4_˙^−^) radical attack. Key steps include N-demethylation, aromatic hydroxylation, and C–N bond cleavage leading to intermediate compounds and eventual mineralization to CO_2_ and H_2_O. Comparative toxicity of intermediates is indicated based on literature-reported ECOSAR and bioassay data.

Including peroxymonosulfate (PMS) and peroxydisulfate (PDS) activation, have recently gained attention for antidepressant degradation due to their ability to generate both sulfate (SO_4_˙^−^) and hydroxyl radicals. Transition metal catalysts (Co^2+^, Fe^3+^, MnO_2_), carbon-based activators, and photocatalytic activation under visible light have been reported to achieve >95% degradation of fluoxetine and citalopram within 30–60 min. Compared to hydroxyl radical systems, sulfate radicals exhibit longer lifetimes and higher selectivity, enabling efficient oxidation even under near-neutral pH conditions. However, metal-ion leaching and secondary sulfate release require careful control. Sulfate radical-based AOPs (SR-AOPs) have emerged as a highly effective alternative to traditional hydroxyl radical (˙OH) processes for the degradation of recalcitrant antidepressants. SO_4_˙^−^ radicals possess a slightly higher oxidation potential (2.5–3.1 V) than ˙OH (1.8–2.7 V) and exhibit greater selectivity toward electron-rich functional groups, such as aromatic rings and tertiary amines, commonly present in fluoxetine, venlafaxine, and amitriptyline.^19^ The activation of persulfate (PMS or PS) can be achieved *via* thermal, UV, transition metal, or electrochemical methods, providing flexibility in operational conditions. Compared to ˙OH, SO_4_˙^−^ often demonstrates higher stability in neutral to slightly alkaline pH and lower scavenging by background water constituents, improving degradation efficiency in real wastewater matrices Fig. 4 illustrates typical degradation pathways of representative antidepressants under SO_4_˙^−^ attack, highlighting N-demethylation, hydroxylation, and aromatic ring cleavage. Overall, SR-AOPs complement ˙OH-based processes and offer distinct advantages in selectivity, stability, and scalability for wastewater treatment applications. Novel piezocatalyst Bi2Fe4O9 nanosheets (BFO NSs) are demonstrated to significantly improve peroxydisulfate (PDS) activation efficiency *via* piezocatalysis, resulting SO_4_˙^−^ and ˙OH as the dominant active species for organic pollutants degradation.^96,97^

#### 3.1.6 Electrochemical advanced oxidation processes (EAOPs)

Combine direct electron transfer reactions with the *in situ* generation of H_2_O_2_ and ˙OH radicals. Boron-doped diamond (BDD), SnO_2_, and PbO_2_ anodes have demonstrated high degradation efficiencies (>90%) for SSRIs such as fluoxetine and venlafaxine. Hybrid electro-Fenton (EF) and photo-electro-Fenton (PEF) systems further enhance oxidation potential by simultaneously producing reactive radicals *via* both electrochemical and photochemical routes. Despite excellent performance, the energy cost (typically 0.8–2.5 kWh g of pollutant removed) remains a limiting factor for industrial applications. Electrochemical Advanced Oxidation Processes (EAOPs) are a class of Advanced Oxidation Processes (AOPs) that utilize electrochemical systems to generate strong oxidants, primarily hydroxyl radicals (˙OH), for the degradation of organic pollutants in water and wastewater. The core principle of EAOPs involves applying an electric current to an electrolytic cell, which promotes the formation of reactive oxidizing species at the electrodes. These species effectively break down complex organic contaminants into more straightforward, less harmful compounds.^98^

The key reactions in EAOPs include the anodic oxidation of water, which generates ˙OH, and the cathodic reduction of oxygen, resulting in the production of hydrogen peroxide (H_2_O_2_). Under specific conditions, H_2_O_2_ can further decompose into ˙OH, enhancing the oxidative capacity of the process. The fundamental reactions are as follows eqn (5)–(7):

Anodic oxidation:

5 H_2_O → OH˙ + H^+^ + e^−^

Cathodic reduction:

6 O_2_ + 2H^+^ + 2e^−^ → H_2_O_2_

7 H_2_O_2_ + e^−^ → OH˙ + OH^−^

These reactions demonstrate the efficiency and adaptability of EAOPs in generating highly reactive species that effectively degrade persistent organic pollutants.^99^

##### 3.1.6.1 Types of electrochemical AOPs

Electrochemical Advanced Oxidation Processes (EAOPs) are classified into several types based on their mechanism of oxidant generation. Each approach has unique advantages and applications in wastewater treatment.

###### 3.1.6.1.1 Anodic oxidation

Anodic oxidation is one of the most widely studied EAOPs, where pollutants are degraded directly at the anode surface or indirectly through the *in situ* generation of hydroxyl radicals (˙OH). The effectiveness of this process largely depends on the electrode material, as it influences the rate of oxidation and the selectivity toward pollutant degradation. Typical anode materials include boron-doped diamond (BDD), platinum (Pt), and lead dioxide (PbO_2_). Among these, BDD electrodes are particularly efficient due to their high overpotential for oxygen evolution, which enhances ˙OH production and minimizes side reactions, such as oxygen gas formation. This makes BDD anodes highly effective for degrading recalcitrant organic contaminants, including pharmaceuticals and industrial dyes.^98^

###### 3.1.6.1.2 Electro-Fenton process

The Electro-Fenton (EF) process integrates electrochemical oxidation with Fenton's chemistry to generate ˙OH. In this method, hydrogen peroxide (H_2_O_2_) is continuously produced at the cathode by reducing dissolved oxygen, while ferrous ions (Fe^2+^) are introduced to catalyze the decomposition of H_2_O_2_ into ˙OH. The key reaction governing this process is represented as follows^100^ (eqn (8)):

8 Fe^2+^ + H_2_O_2_ → Fe^3+^ + OH^−^ + OH˙

The electro-Fenton process is highly efficient for completely mineralizing organic pollutants, converting them into CO_2_ and water. This method is particularly advantageous for treating wastewater containing persistent organic pollutants, including pesticides, pharmaceuticals, and endocrine-disrupting compounds. Additionally, the *in situ* generation of oxidants eliminates the need for external chemical dosing, making the process environmentally friendly and cost-effective.^101^

###### 3.1.6.1.3 Photoelectrochemical oxidation

Photoelectrochemical (PEC) oxidation combines electrochemical and photochemical techniques to enhance pollutant degradation. This method employs a photoactive electrode, typically titanium dioxide (TiO_2_), which generates electron–hole pairs upon UV or visible light irradiation. The photogenerated holes (h+) oxidize water molecules to form ˙OH, while the electrons reduce oxygen to form H_2_O_2_, further boosting the oxidation process.^102^ The synergistic interaction between light energy and electrochemical activation significantly improves pollutant degradation efficiency compared to conventional electrochemical oxidation. This method is particularly effective for removing emerging contaminants, such as antibiotics and persistent dyes, from wastewater.

EAOPs exhibit several advantages over conventional wastewater treatment methods:

High oxidation potential: the *in situ* generation of ˙OH ensures rapid and effective degradation of recalcitrant organic pollutants.

Operational flexibility: the process parameters, such as applied current, voltage, and electrode material, can be adjusted to optimize pollutant removal efficiency.

Compatibility with other treatment methods: EAOPs can be integrated with biological filtration, adsorption, or membrane processes to enhance overall wastewater treatment performance.^103–105^

Eco-friendly approach: since EAOPs generate oxidants electrochemically, they reduce the need for hazardous chemical additions, minimizing the risk of secondary pollution. Furthermore, the degradation by-products are generally less toxic than those produced by conventional oxidation methods.

Recent studies have focused on optimizing EAOPs for large-scale applications in wastewater treatment. A comprehensive review by Oturan and Brillas highlighted the effectiveness of anodic oxidation and electro-Fenton processes in degrading persistent organic pollutants, emphasizing the importance of optimizing electrode materials and operational parameters.^99^ Similarly, Feijoo *et al.*, provided an in-depth analysis of oxidative species generation mechanisms in EAOPs, shedding light on the strengths and limitations of different approaches.^98^ Case studies have demonstrated the successful application of EAOPs for removing pharmaceuticals from wastewater. For example, Sires *et al.* reported significant reductions in antidepressant concentrations using EAOPs, confirming their potential for municipal and industrial wastewater treatment.^106^ Additionally, research on EAOPs has underscored their role in improving water quality and ensuring compliance with environmental regulations.^107^

EAOPs represent a promising and versatile technology for degrading organic pollutants in wastewater. Their high oxidation efficiency, adaptability, and environmental benefits make them a superior alternative to conventional treatment methods. Continued advancements in electrode materials, reactor design, and process optimization will further enhance the effectiveness and applicability of EAOPs in environmental remediation.

## 4 Chemical mechanisms of antidepressant degradation under AOPs

Advanced Oxidation Processes (AOPs) are powerful chemical technologies capable of degrading recalcitrant pharmaceutical pollutants through the generation of highly reactive radical species. These radicals, primarily hydroxyl (˙OH) and sulfate (SO_4_˙^−^) exhibit strong oxidation potentials and can non-selectively attack complex organic structures such as those of antidepressants. The degradation of antidepressants by AOPs involves radical generation, electrophilic attack on functional moieties, intermediate formation, and eventual mineralization into CO_2_, H_2_O, and inorganic ions.^108^ The degradation pathways of antidepressants typically involve multiple oxidative steps, including hydroxylation, demethylation, and aromatic ring cleavage. For instance, venlafaxine, a widely used antidepressant, undergoes hydroxylation at the aromatic ring, followed by ring cleavage, forming smaller organic acids and, ultimately, complete mineralization. Similar pathways have been observed for fluoxetine and bupropion, where oxidation leads to the breakdown of their molecular structures into biodegradable intermediates.^51^ The degradation of antidepressants through AOPs primarily proceeds *via* hydroxyl (˙OH) and sulfate (SO_4_˙^−^) radical pathways. Both radicals initiate electrophilic attacks on electron-rich aromatic and amine groups, triggering N-demethylation, hydroxyl substitution, and aromatic ring cleavage. For instance, fluoxetine undergoes stepwise transformation into norfluoxetine, followed by hydroxylated and carboxylated intermediates, ultimately yielding CO_2_ and H_2_O. However, partial oxidation can generate intermediates with transient toxicity, such as phenolic or amide derivatives. Toxicity assessments based on ECOSAR and Daphnia magna assays indicate that sulfate radical-based AOPs generally produce fewer toxic byproducts due to deeper mineralization efficiency. Thus, understanding radical selectivity and degradation sequence is essential for designing sustainable treatment systems for antidepressant-contaminated water as shown in Fig. 4.

### 4.1 Radical generation and reactivity

Hydroxyl radicals (*E*° = +2.8 V) are generated through processes such as Fenton (Fe^2+^/H_2_O_2_), photo-Fenton (Fe^2+^/H_2_O_2_/UV), UV/H_2_O_2_, and photocatalytic oxidation (TiO_2_/UV). In contrast, sulfate radicals (*E*° = +2.6 V) are formed *via* activation of persulfate (S_2_O_8_^2−^) or peroxymonosulfate (HSO_5_^−^) through heat, UV, transition metals, or carbon-based catalysts. Hydroxyl radicals are highly non-selective, reacting with most organic molecules at diffusion-controlled rates (10^8^–10^10^ M^−1^ s^−1^), whereas sulfate radicals exhibit greater selectivity toward electron-rich sites and aromatic amines^109^

9 Fe^2+^ + H_2_O_2_→ Fe^3+^ + OH^−^ + OH˙

10 S_2_O_8_^−2^ + heat/UV/Fe^+2^ → 2SO_4_˙^−^

The generated ˙OH then attacks antidepressant molecules, breaking them down into intermediates, which undergo further oxidation until they are completely mineralized.

### 4.2 Photoelectrochemical oxidation

This method enhances ˙OH production by integrating electrochemical and photochemical processes. UV or visible light irradiates a photoactive electrode, typically TiO_2_, generating electron–hole pairs. The holes oxidize water to form, while the electrons reduce oxygen to form H_2_O_2_. This synergistic mechanism significantly improves pollutant degradation efficiency.^110^ Hydroxyl radicals (*E*° = +2.8 V) attack electron-rich aromatic rings and aliphatic amine groups, initiating N-demethylation and hydroxylation reactions. Sulfate radicals (*E*° = +2.6 V), generated *via* persulfate or peroxymonosulfate activation, exhibit higher selectivity and promote electron-transfer mechanisms, often resulting in ring-opening or defluorination processes in fluorinated antidepressants such as fluoxetine.

### 4.3 Kinetic and computational perspectives

The degradation kinetics of antidepressants by AOPs typically follow pseudo-first-order behavior:

11 ln(*C*_0_/*C*_*t*_) = *K*_a_*t*

where *K*_a_ is the apparent rate constant dependent on oxidant dose, catalyst concentration, and pH. Comparative studies have shown that UV/PS and electro-Fenton systems achieve rate constants up to three times higher than conventional UV/H_2_O_2_ systems.

Density Functional Theory (DFT) simulations further reveal that ˙OH radicals preferentially attack aromatic sites with high electron density (HOMO regions), while SO_4_˙^−^ radicals target amine and ether groups (LUMO regions). This explains the experimentally observed dominance of N-demethylation under persulfate activation and hydroxylation under photocatalytic oxidation.^111,112^

Overall, AOP driven degradation of antidepressants proceeds through multi step radical oxidation, involving hydroxylation, dealkylation, and aromatic cleavage. The reaction route and mineralization efficiency are dictated by the radical species, oxidant activation pathway, and structural features of the antidepressant molecule.

These mechanistic insights not only advance understanding of pollutant transformation chemistry but also guide the rational design of efficient, sustainable AOP systems for antidepressant removal.

### 4.4 AOP integration for enhanced efficiency

**Fig. 5:**
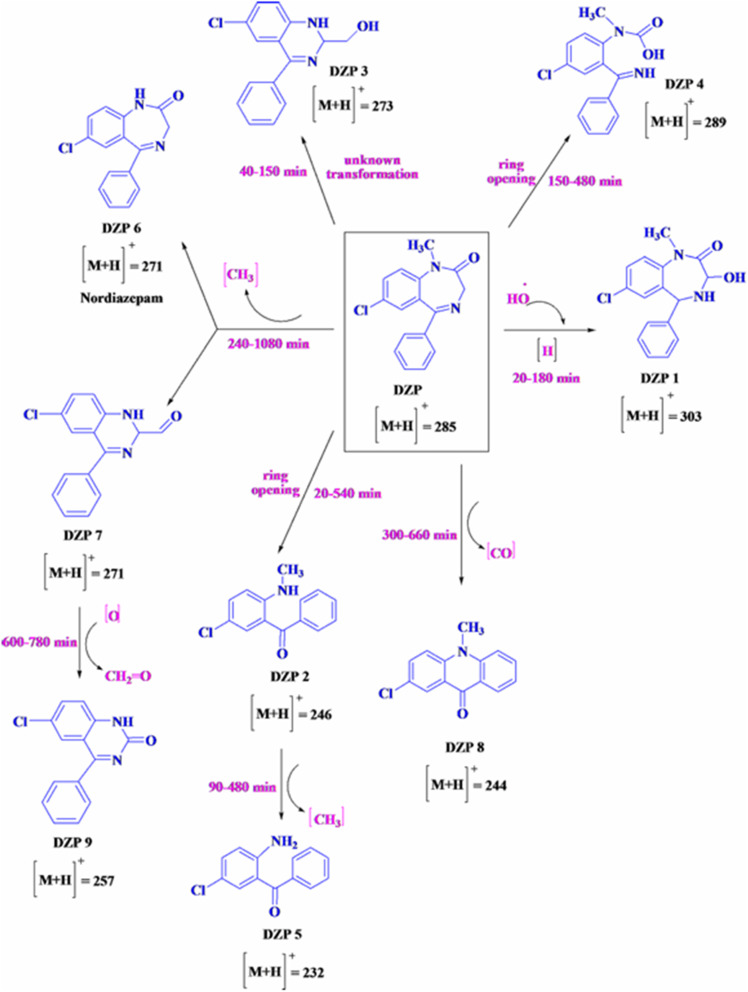
Suggested pathway for the photodegradation of diazepam, reproduced from ref. 115, with permission from Longdom, Copyright © 2014.

AOPs can be effectively integrated with biological treatments or adsorption techniques to enhance the removal of pollutants. Biological therapies facilitate the degradation of oxidation-resistant intermediates that may form during AOP treatment, thereby promoting complete mineralization. Additionally, combining AOPs with adsorption processes (*e.g.*, activated carbon or biochar) can improve efficiency by concentrating pollutants before oxidative degradation, as illustrated in Fig. 5.^113,114^

#### 4.4.1 Environmental and practical advantages

AOPs offer a sustainable and environmentally friendly approach to wastewater treatment. Unlike conventional methods, they do not require harmful chemical additives, as oxidants are generated *in situ*, thereby reducing the risk of secondary pollution. Moreover, the by-products of AOPs are generally less toxic than those produced by traditional treatments, making these processes highly promising for removing pharmaceuticals and other emerging contaminants from wastewater.^116,117^

Researchers continuously advance AOP technology to optimize process parameters and integrate hybrid treatment strategies, ensuring more efficient and cost-effective wastewater remediation.

## 5 Hybrid AOP systems and synergistic mechanisms

The integration of Advanced Oxidation Processes (AOPs) with complementary treatment technologies has recently gained attention as a sustainable approach for improving pollutant degradation efficiency and minimizing operational costs. Hybrid AOP systems combine the radical-based oxidation power of AOPs with the selectivity or biodegradability enhancement offered by biological, adsorptive, or membrane-based methods. Such integration not only enhances overall removal efficiency but also mitigates secondary pollution and facilitates continuous operation under practical wastewater treatment conditions.^62,118^

AOPs coupled with biological treatment have demonstrated remarkable synergy for the degradation of pharmaceutical contaminants, including antidepressants. In these systems, AOPs act as a pre-oxidation step to transform persistent compounds such as fluoxetine, venlafaxine, and citalopram into more biodegradable intermediates, thus increasing the biochemical oxygen demand (BOD_5_/COD) ratio of the wastewater. For example, UV/persulfate pre-oxidation enhanced the biodegradability of fluoxetine-contaminated water by nearly 70%, facilitating subsequent microbial mineralization.^119,120^ The hybrid AOP–bio treatment thus achieves both rapid oxidation and cost-effective mineralization through sequential oxidation–biodegradation pathways.

AOP–adsorption coupling offers another promising strategy. Hybrid catalysts such as Fe_3_O_4_@graphene and g-C_3_N_4_/CuO nanocomposites integrate adsorption and oxidation functionalities, allowing for effective concentration of pollutants on the catalyst surface and simultaneous degradation by *in situ* generated radicals. These systems improve mass transfer, reduce scavenging effects, and enhance the recyclability of heterogeneous catalysts. Recent studies reported >95% removal of venlafaxine and sertraline using visible-light-driven g-C_3_N_4_–based composites.^121,122^

AOP–membrane hybrid systems combine photocatalytic or electrochemical AOPs with membrane separation to achieve simultaneous degradation and filtration. Membranes coated with photocatalysts such as TiO_2_, ZnO, or BiVO_4_ enable dynamic removal of antidepressants while preventing catalyst leaching and fouling. These systems are particularly attractive for continuous-flow treatment configurations, where pollutants are oxidized before permeate recovery.^123,124^

The synergistic mechanisms in hybrid AOP systems primarily arise from (i) enhanced mass transfer between pollutants and reactive radicals, (ii) *in situ* production of biodegradable intermediates facilitating microbial degradation, and (iii) regeneration of surface-bound radicals on catalyst interfaces. These hybrid processes collectively improve mineralization efficiency, lower energy consumption, and extend catalyst lifespan, demonstrating a practical and green pathway for large-scale antidepressant removal.^18,125,126^

## 6 Application of AOPs for antidepressant removal

### 6.1 Efficiency of different AOPs

Various AOPs have been investigated for their effectiveness in degrading antidepressants in wastewater Table 3. These processes utilize highly reactive oxidative species, such as hydroxyl radicals (˙OH), to break down complex pharmaceutical compounds into more straightforward, less toxic by-products.

**Table 3:** Removal efficiency of various AOP methods for antidepressant degradation under different reaction conditions

Wastewater treatment method	Specific conditions	Antidepressant removed	Efficiency of removal (%)	Reference
Advanced oxidation processes	UVA irradiation	DOX, VEN	30–40	134
	TiO_2_ (0.5 g L^−1^), pH 7, 120 min, TiO_2_ (0.8 g L^−1^), pH 6, 150 min	FLU	15	135
	UVA + UVB irradiation	DES	50	136
	TiO_2_ (0.8 g L^−1^), pH 6, 150 min			
	UVC irradiation	AMI, CLO	88–100	137
	TiO_2_ (0.3 g L^−1^), pH 7, 60 min	TRI	92	138
	O_3_ + TiO_2_ + UVA [O_3_] = 10 mg L^−1^, TiO_2_ 0.6 g L^−1^, pH 7, 90 min	FLU	50	135
	O_3_ + H_2_O_2_ (PEROXONE) + UVA [O_3_] = 10 mg L^−1^, [H_2_O_2_] = 5 mM, pH 6.5, 90 min	FLU	70	135
	O_3_ + TiO_2_ + H_2_O_2_ + UVA	FLU	97	135
	Gamma (*γ*) radiation	SER, CIT	80–100	139
	5 kGy dose, pH 7, 30 min			
	Electro-oxidation using BDD anode 0 mA cm^−2^, pH 6, 160 min	AMI	76	140
	Electro-Fenton (EF) [Fe^2+^] = 0.2 mM, [H_2_O_2_] = 10 mM, pH 3, 160 min	AMI	78	140
	Photoelectro-Fenton (PEF) [Fe^2+^] = 0.2 mM, [H_2_O_2_] = 10 mM, pH 3, UVA irradiation, 160 min	AMI	95	140

Several studies have demonstrated the efficiency of different AOPs for antidepressant degradation. For instance, TiO_2_ nanoparticles, when exposed to UV light, were used to degrade fluoxetine, achieving an 85% removal efficiency after 120 minutes of treatment.^127^ Similarly, ozonation was employed to treat wastewater containing venlafaxine and citalopram, resulting in removal efficiencies of 90% and 82% after 60 minutes, respectively.^128,129^ Electrochemical oxidation has also shown promising results, with a study reporting the removal of 96% fluoxetine from synthetic wastewater after 160 minutes of electrochemical treatment.^130^ The UV/H_2_O_2_ process effectively degraded venlafaxine, with a removal efficiency of 87% within 60 minutes.^131^ Additionally, ultrasonic waves were utilized for the degradation of amitriptyline in aqueous solutions, achieving a 75% removal efficiency after 120 minutes of sonication.^132^ Finally, ZnO nanoparticles under UV light were tested for citalopram removal, achieving 75% degradation within 120 minutes of treatment.^133^

This comparative data highlights the effectiveness of various AOPs, with photoelectro-Fenton (PEF) demonstrating the highest efficiency (95%) for amitriptyline removal. Integrating multiple oxidative techniques, such as ozonation combined with TiO_2_ and H_2_O_2_ under UVA irradiation, also significantly enhances degradation efficiency, reaching up to 97% for fluoxetine.

These findings underscore the potential of AOPs as efficient wastewater treatment strategies for removing pharmaceutical pollutants, particularly antidepressants.

#### 6.1.1 Factors affecting efficiency

##### 6.1.1.1 Effect of pH

The pH of a solution plays a crucial role in determining the efficiency of AOPs for removing antidepressants, as it directly influences the generation and stability of reactive species, such as hydroxyl radicals, which are essential for degradation. Studies have demonstrated that optimal pH conditions vary depending on the specific AOP and antidepressant being treated. Hollman *et al.* found that the degradation efficiency of venlafaxine using the UV/H_2_O_2_ process was highest at pH 3, with efficiency declining as the pH increased.^141^ Similarly, Racovita and Ciuca observed that the removal efficiency of tricyclic antidepressants (TCAs) significantly decreased at pH values above 7 due to partial deprotonation and reduced solubility.^3^ Waris and Farooqi reported a sharp decline in the degradation efficiency of tetracycline, from over 90% at pH 3 to just 50% at pH 5.83, emphasizing the impact of pH variations on removal rates.^142^ In contrast, Li *et al.* demonstrated that the UV/chlorine-BAC process was most effective at pH 7 for amitriptyline removal, as the combination of UV and chlorine exhibited optimal performance under neutral conditions.^143^ Furthermore, Aghaeinejad-Meybodi *et al.* highlighted that catalytic ozonation of fluoxetine showed maximum efficiency at neutral pH, with significant reductions observed under both acidic and alkaline conditions.^144^ These findings collectively underscore the need to optimize pH conditions to enhance the effectiveness of AOPs in treating antidepressant contaminants in water.^145,146^ The solution pH plays a pivotal role in determining the oxidation efficiency of AOPs by influencing radical generation, catalyst surface charge, and pollutant ionization state. In Fenton and photo-Fenton processes, acidic conditions (typically pH 2.5–3.0) favor the formation of hydroxyl radicals (˙OH) through the Fe^2+^/H_2_O_2_ redox cycle. Under neutral or alkaline conditions, Fe^3+^ tends to precipitate as Fe(OH)_3_, reducing the catalytic activity and radical yield. In contrast, persulfate-based AOPs often exhibit higher performance under near-neutral to mildly alkaline pH (6–9) because base activation facilitates the generation of sulfate (SO_4_˙^−^) and hydroxyl radicals *via* hydrolysis and electron-transfer reactions. Similarly, in photocatalytic systems (*e.g.*, TiO_2_ or doped metal oxides), alkaline pH enhances surface hydroxylation and promotes the formation of reactive ˙OH through hole–OH^−^ interactions. Therefore, the optimal pH for each AOP is a balance between catalyst stability, radical lifetime, and pollutant reactivity, which collectively govern the degradation kinetics of antidepressants.^57,147^

##### 6.1.1.2 Effect of catalyst dosage

The amount of catalyst used in AOPs plays a crucial role in determining the degradation efficiency of antidepressants, as it directly influences the generation of reactive species that enhance oxidation. Studies have shown that increasing the dosage of nano-boehmite and nano-γ-alumina catalysts significantly improves the degradation of fluoxetine in water.^144^ Similarly, higher doses of hydrogen peroxide in UV/H_2_O_2_ processes reduce the required basin volume but increase operating costs.^141^ In catalytic ozonation, raising ozone dosages has been found to lower both operating costs and basin volume, making the process more efficient.^141^ Research on pharmaceutical degradation further indicates that the optimal catalyst dosage varies depending on the specific AOP and the target compound, highlighting the need for process-specific optimization.^3^ Additionally, photocatalytic degradation studies reveal that increasing catalyst dosage enhances efficiency up to a certain threshold, beyond which further increases do not yield significant improvements.^144^ These findings emphasize the importance of carefully selecting and optimizing catalyst amounts to maximize efficiency while maintaining cost-effectiveness in antidepressant removal.

##### 6.1.1.3 Effect of reaction time

The duration of the reaction is a critical factor in achieving complete degradation of antidepressants in AOPs, with longer reaction times generally leading to higher removal efficiencies. However, the optimal duration varies depending on the specific process and treated compound. Studies have shown that in UV/chlorine-BAC treatment, an optimal reaction time of 15 minutes of UV exposure followed by 30 minutes of BAC treatment resulted in 98.5% removal of amitriptyline.^143^ Similarly, extended reaction times in UV/H_2_O_2_ processes have significantly enhanced degradation efficiency.^67^ In electrochemical oxidation, a reaction time of up to 60 minutes was ultimately required to mineralize pharmaceutical contaminants.^72^ Ozone-based AOPs also demonstrated increased efficiency with longer reaction durations, though diminishing returns were observed beyond a certain point.^3^ Additionally, research on the photocatalytic degradation of fluoxetine highlighted that optimal reaction times depend on factors such as initial concentration and catalyst dosage, emphasizing the need for process-specific adjustments.^144^ These findings underscore the importance of optimizing reaction duration to strike a balance between efficiency and resource utilization in antidepressant removal.^148–153^ Degradation kinetics typically follow pseudo-first-order behavior, with apparent rate constants dependent on oxidant dosage and radical availability. For instance, fluoxetine degradation by UV/PS systems proceeds 1.6 times faster than by UV/H_2_O_2_, attributed to the longer lifetime and higher selectivity of sulfate radicals.

### 6.2 Byproducts and toxicity

In wastewater treatment using AOPs, the degradation of antidepressants such as fluoxetine, venlafaxine, and citalopram involves complex pathways that break down the parent compounds into various intermediate byproducts. These transformations typically occur through mechanisms such as hydroxylation, demethylation, oxidation, and cleavage of aromatic rings, which can lead to the formation of compounds that are sometimes more toxic than the original antidepressants. The persistence of these byproducts in treated wastewater poses significant ecological concerns, particularly for aquatic ecosystems, as they can accumulate in sediments and biofilms, prolonging their environmental impact.^43^

Fluoxetine, a widely prescribed selective serotonin reuptake inhibitor (SSRI), undergoes degradation during AOP treatment primarily through hydroxylation of the aromatic ring, N-demethylation, and oxidative cleavage of the amine group.^1^ This process generates several key byproducts, including norfluoxetine and fluoxetine-N-oxide, along with multiple hydroxylated derivatives. Among these, norfluoxetine, the primary metabolite, has been shown to exhibit greater endocrine-disrupting potential in aquatic organisms, affecting reproductive behavior and developmental functions.^1^ Fluoxetine-N-oxide, another significant byproduct, has been linked to behavioral changes and reduced survival rates in aquatic species, emphasizing the ecological risks associated with its persistence in treated wastewater.^1^

Similarly, venlafaxine, a commonly prescribed serotonin-norepinephrine reuptake inhibitor (SNRI), undergoes oxidative degradation pathways during AOP treatment, leading to the formation of major intermediates such as *O*-desmethylvenlafaxine, N-desmethylvenlafaxine, and hydroxylated derivatives. *O*-desmethylvenlafaxine, known for its increased toxicity, has been linked to reproductive impairments, including lower fertilization rates and abnormal embryonic development in aquatic organisms.^3^ N-desmethylvenlafaxine also exhibits heightened toxicity, impairing the growth, survival, and overall health of aquatic species exposed to treated effluents.^3^ Both compounds demonstrate increased persistence in the environment, raising concerns about their long-term ecological impacts.

Citalopram, another frequently detected antidepressant in wastewater, undergoes degradation through demethylation, hydroxylation, and ring cleavage, producing intermediates such as desmethylcitalopram and citalopram-N-oxide. Desmethylcitalopram has been shown to disrupt endocrine functions in aquatic organisms, causing reproductive abnormalities and developmental delays.^3^ Citalopram-N-oxide, another significant byproduct, has been associated with behavioral changes and increased mortality in fish and invertebrates, further emphasizing the need for effective wastewater treatment strategies to mitigate these risks.^3^

Such toxic byproducts in treated wastewater can have profound ecotoxicological impacts, even at trace concentrations.^43^ These compounds can bioaccumulate in aquatic organisms, leading to hormonal imbalances, reproductive dysfunction, and population declines.^43^ For instance, exposure to norfluoxetine has been linked to delayed spawning and reduced fertility in fish. At the same time, venlafaxine byproducts have been associated with impaired embryonic development and growth inhibition in aquatic invertebrates.^1,3^ Moreover, the persistence of these intermediates in the environment can lead to their accumulation in sediments and biofilms, further extending their impact across trophic levels.^43^

The ecological impact of treated wastewater containing residual antidepressants and their degradation byproducts is a significant environmental concern. Despite undergoing advanced wastewater treatment processes, trace amounts of these compounds often persist in effluents, ultimately entering aquatic environments. These residual contaminants can hurt marine organisms, disrupt the ecological balance, and lead to long-term environmental consequences.^154^

#### 6.2.1 impact on aquatic organisms

One of the most concerning effects of treated wastewater containing antidepressants and their by-products is the impact on aquatic organisms. Even at low concentrations, these compounds can interfere with the physiological and behavioral functions of aquatic species.^155^

• Behavioural changes: antidepressants such as fluoxetine, venlafaxine, and citalopram, as well as their degradation products, can alter the behavior of fish, amphibians, and invertebrates. For example, exposure to fluoxetine has been linked to increased boldness, altered feeding habits, and disrupted reproductive behavior in fish.^156^ Norfluoxetine, a key by-product, has been associated with increased aggression and abnormal social interactions, further affecting population dynamics.^156^

• Reproductive effects: antidepressant residues and their toxic by-products can impair the reproductive capabilities of aquatic organisms. Venlafaxine and its degradation products, such as desmethylvenlafaxine, have been linked to reduced egg production, lower fertilization rates, and delayed hatching in fish and amphibians.^157^ These reproductive impairments, observed even at environmentally relevant concentrations, threaten aquatic biodiversity significantly.^157^

• Developmental toxicity: aquatic organisms' developmental stages are particularly vulnerable to antidepressant exposure. Citalopram and its byproducts, such as desmethyl citalopram and citalopram-N-oxide, have been linked to developmental abnormalities in fish and amphibians.^158^ Impaired embryonic development, malformations, and reduced larval survival rates have been reported, posing long-term risks to population structures and ecosystem stability.^158^

#### 6.2.2 Bioaccumulation and biomagnification

The bioaccumulation and biomagnification of antidepressants and their byproducts further exacerbate the ecological impact of treated wastewater.

• Bioaccumulation: antidepressants and their degradation intermediates can accumulate in the tissues of aquatic organisms, leading to higher internal concentrations than those found in the surrounding water.^159^ Fish, mollusks, and crustaceans exposed to contaminated water can accumulate these compounds in their muscles, liver, and brain tissues. For instance, norfluoxetine has been detected at elevated levels in fish tissues, raising concerns about long-term impacts on aquatic health and food chain dynamics.^160^

• Biomagnification: as these compounds move up the food chain, their concentrations can increase through biomagnification.^159^ Predatory species, such as larger fish and aquatic birds, may accumulate higher levels of antidepressants and their byproducts by consuming contaminated prey. This accumulation can lead to toxic effects in top predators, including hormonal imbalances, reproductive dysfunction, and compromised immune systems, highlighting the urgent need for advanced wastewater treatment strategies.^159^

#### 6.2.3 Ecosystem disruption

Beyond individual organism-level effects, residual antidepressants in treated wastewater can disrupt entire aquatic ecosystems by affecting primary producers, microbial communities, and nutrient cycles.

• Algal blooms: antidepressants can alter nutrient dynamics in aquatic environments, promoting algae growth, including harmful algal species.^161^ This rapid proliferation, driven by nutrient imbalances, can lead to hypoxic conditions, threatening fish and other aquatic organisms.

• Microbial communities: antidepressants and their byproducts can also affect the composition and function of microbial communities in aquatic environments. Alterations in microbial diversity, inhibition of beneficial bacteria, and promotion of antibiotic-resistant strains can disrupt nutrient cycling processes, such as nitrogen fixation and organic matter decomposition, ultimately affecting ecosystem resilience.^162^

The persistence of antidepressant residues and their byproducts in aquatic environments can lead to long-term ecological consequences. Chronic exposure to low concentrations of these compounds can result in population declines, biodiversity loss, and shifts in ecosystem dynamics. Sensitive species may be particularly vulnerable, while tolerant species may become more dominant, altering community structures. Moreover, the potential for cross-contamination between aquatic and terrestrial ecosystems further underscores the need for comprehensive wastewater management strategies to mitigate the ecological risks associated with antidepressant residues.

To address the ecological concerns associated with treated wastewater containing antidepressants and their byproducts, comprehensive treatment strategies are essential to ensure complete mineralization and prevent environmental release. Advanced treatment methods, such as combined AOPs, biological post-treatment, and membrane filtration, can significantly enhance the removal efficiency of these compounds.^163^ Implementing such strategies reduces the persistence of toxic byproducts and minimizes their potential to harm aquatic organisms and disrupt ecosystems. Furthermore, continuous monitoring and ecotoxicological assessments are crucial for evaluating the long-term impacts of these residues, guiding the development of sustainable wastewater treatment approaches. Ultimately, a multifaceted approach that integrates effective treatment, vigilant monitoring, and thorough ecological evaluation is vital to safeguarding aquatic environments from the adverse effects of antidepressant contamination. Table 4 illustrates the AOPs for antidepressant removal.

**Table 4:** Overview of AOPs for antidepressant removal

Antidepressant	AOP method	Key findings	Successful applications	Limitations	References
Venlafaxine	UV/h?o_2_	UV/H_2_O_2_ and UV/O_3_ were cost-competitive; UV alone was not viable	Effective in reducing venlafaxine levels in wastewater	High operating costs for UV treatment; requires optimization of O_3_ dosage	141
	UV/O_3_				
	O_3_				
Various TCAs	Ozone	Ozone and UV are effective in degrading TCAs; electrochemical methods also show promise	Successfully reduced TCA concentrations in wastewater	High energy consumption is associated with UV and electrochemical methods	3
	UV				
	Electrochemical				
Fluoxetine	Catalytic ozonation (nano-boehmite, nano-γ-alumina)	Catalytic ozonation significantly improved degradation efficiency	Enhanced removal of fluoxetine in water treatment	It requires specific catalysts, and there is potential for catalyst deactivation	144
Amitriptyline	UV/Chlorine-BAC	Optimal pH around 7; achieved 98.5% removal with 15 minutes of UV and 30 minutes of BAC.	Effective in drinking water treatment	Requires precise control of pH and reaction time	143
Fluoxetine	Ozonation	Combining TiO_2_/O_3_/H_2_O_2_ with UV showed the highest efficiency	Effective in removing fluoxetine from aqueous solutions	Complex setup; high operational costs	135
	Peroxone				
	TiO_2_/O_3_				
	TiO_2_/O_3_/H_2_O_2_				

## 7 Challenges and limitations

### 7.1 Operational challenges

#### 7.1.1 High energy and chemical requirements

Advanced oxidation processes (AOPs) have emerged as promising technologies for degrading persistent organic pollutants, including antidepressants, in wastewater. Antidepressants, such as selective serotonin reuptake inhibitors (SSRIs) and tricyclic antidepressants (TCAs), are increasingly detected in aquatic environments due to their widespread use and incomplete removal by conventional wastewater treatment plants.^164^ These pharmaceuticals can harm marine ecosystems and human health, even at low concentrations. AOPs, which generate highly reactive hydroxyl radicals (˙OH), effectively break down these recalcitrant compounds into less harmful byproducts. However, the high energy and chemical requirements of AOPs pose significant challenges to their widespread implementation.^165^

AOPs such as photocatalysis, ozonation, Fenton processes, and UV-based systems rely on generating ˙OH radicals, which non-selectively oxidize organic pollutants. While these methods are highly effective, they often demand substantial energy inputs. For instance, UV-based AOPs require high-intensity UV lamps, which consume significant amounts of electricity.^166^ Photocatalysis, which involves semiconductors like TiO_2_, also necessitates UV or visible light sources, adding to the energy burden. Ozonation, another widely studied AOP, requires the production of ozone, an energy-intensive process that involves high-voltage electrical discharges. The energy requirements of these processes can limit their scalability and economic feasibility, particularly in large-scale wastewater treatment applications.^167^

In addition to energy demands, AOPs often require chemicals, which can increase operational costs and environmental impacts. For example, the Fenton process relies on adding hydrogen peroxide (H_2_O_2_) and iron catalysts to generate ˙OH radicals. While effective, the continuous supply of H_2_O_2_ and managing iron sludge generated during the process add to the chemical and operational complexity. Similarly, ozonation requires the production and storage of ozone, which can be hazardous and costly. The need for pH adjustment in some AOPs, such as the Fenton process, further increases chemical consumption and complicates the treatment process.^164^

Recent studies have explored strategies to mitigate the high energy and chemical requirements of AOPs. For instance, integrating renewable energy sources, such as solar energy, into photocatalytic systems has shown promise in reducing energy consumption. The development of more efficient catalysts, such as doped TiO_2_ or composite materials, has also been investigated to enhance the degradation efficiency of antidepressants while minimizing energy and chemical inputs. Additionally, hybrid AOPs, which combine multiple oxidation processes, have been proposed to improve overall efficiency and reduce costs. Despite these advancements, the high energy and chemical requirements of AOPs continue to be a significant barrier to their widespread adoption. Future research should focus on optimizing process parameters, developing cost-effective catalysts, and integrating renewable energy sources to improve the sustainability of AOPs for removing antidepressants from wastewater. Addressing these challenges will be crucial for successfully implementing AOPs in real-world wastewater treatment scenarios.^168^

#### 7.1.2 Catalyst deactivation or fouling

Catalyst deactivation in AOPs can occur through several mechanisms, including poisoning, fouling, thermal degradation, and leaching of active sites. In the removal of antidepressants, fouling is a predominant issue due to the complex nature of these compounds and their transformation products. Antidepressants, such as selective serotonin reuptake inhibitors (SSRIs) and tricyclic antidepressants (TCAs), often contain aromatic rings and functional groups that can adsorb onto catalyst surfaces, leading to the formation of polymeric by-products or coke. These by-products block active sites, reducing the catalyst's ability to generate reactive species and degrade pollutants.^169^

Fouling is particularly problematic in heterogeneous AOPs, such as photocatalysis and Fenton-based processes, where solid catalysts like TiO_2_ or iron oxides are used. For instance, TiO_2_ photocatalysts are prone to deactivation due to the accumulation of organic intermediates on their surfaces, reducing photocatalytic activity and increasing energy consumption. Similarly, iron-based catalysts can be deactivated in Fenton processes by precipitation of iron hydroxides or the formation of stable complexes with organic ligands, limiting their reusability.^170^ The presence of co-contaminants in wastewater further exacerbates catalyst deactivation. Natural organic matter (NOM), inorganic ions, and other pharmaceuticals can compete for reactive sites or scavenge radicals, reducing the overall efficiency of AOPs. For example, chloride ions, commonly found in wastewater, can react with ˙OH to form less reactive chlorine radicals, while NOM can adsorb onto catalyst surfaces, leading to fouling.

Several strategies have been proposed to address these challenges. Catalyst regeneration through thermal, chemical, or physical methods can restore activity, but these approaches are often energy-intensive and may not be feasible for large-scale applications. Alternatively, developing robust catalysts with enhanced resistance to fouling, such as doped or composite materials, has shown promise. For instance, doping TiO_2_ with nitrogen or carbon has been reported to improve its photocatalytic activity and stability under visible light. Similarly, the use of magnetic catalysts in Fenton processes facilitates easy separation and reuse, thereby reducing the risk of deactivation.^171^

### 7.2 Economic feasibility

#### 7.2.1 Cost of scaling up AOPs for real-world applications

Several factors, including energy consumption, chemical requirements, reactor design, and maintenance, influence the cost of scaling up AOPs. For instance, photocatalytic processes using titanium dioxide (TiO_2_) or other semiconductors require UV light sources, which are energy-intensive and contribute to high operational costs.^172^ Similarly, while effective, ozonation demands significant energy for ozone generation and poses safety concerns due to the handling of toxic gases. The Fenton process, which relies on iron catalysts and hydrogen peroxide, incurs chemical procurement costs and sludge management costs. These factors collectively increase the overall cost of implementing AOPs at a large scale.^128^

Energy consumption is a significant cost driver in AOPs. For example, UV-based systems require continuous electricity to power lamps, and their efficiency decreases with scaling due to issues like light penetration and reactor geometry. Hybrid systems, such as UV/H_2_O_2_ or photo-Fenton, have shown improved degradation efficiencies but at the expense of higher energy and chemical inputs. Additionally, the need for advanced reactor designs to optimize mass transfer and light utilization further escalates capital and operational expenditures. Chemical costs also play a significant role in the economic feasibility of AOPs. Hydrogen peroxide, a standard reagent in many AOPs, is expensive and requires careful handling and storage. Using catalysts, such as iron or titanium dioxide, adds to the material costs, and their recovery or regeneration can be challenging in large-scale applications. Moreover, the formation of toxic by-products during AOPs may necessitate additional treatment steps, further increasing costs.^118^

Despite these challenges, recent advancements have aimed to reduce the cost of AOPs. For instance, developing solar-driven photocatalytic systems leverages renewable energy to minimize electricity consumption. Similarly, heterogeneous catalysts and immobilized systems have shown potential for reducing chemical usage and improving reusability. However, these innovations are still in the early stages of implementation and require further research to demonstrate their viability at scale.^173^

### 7.3 Knowledge gaps

#### 7.3.1 Limited studies on long-term environmental impacts

One of the primary challenges in assessing the long-term environmental impacts of AOPs is the transformation of parent compounds into intermediate by-products. Although AOPs can degrade antidepressants, the resulting transformation products (TPs) may retain biological activity or exhibit unforeseen toxicity. For instance, studies have shown that the degradation of fluoxetine *via* AOPs can yield TPs such as norfluoxetine, which has been reported to exhibit similar or even higher toxicity than the parent compound.^174^ Similarly, the ozonation of citalopram has been found to produce TPs with unknown ecological effects. These findings highlight the need for comprehensive toxicological assessments of TPs generated during AOP treatment.^175^

Another critical concern is the potential for AOPs to alter the physicochemical properties of wastewater, which could have cascading effects on aquatic ecosystems. For example, generating reactive oxygen species (ROS) during AOPs may lead to oxidative stress in marine organisms, even at low concentrations. Additionally, releasing metal catalysts, such as iron or titanium dioxide nanoparticles, from certain AOPs could pose risks to aquatic life and contribute to long-term environmental contamination. Despite these risks, few studies have investigated these catalysts' persistence and bioaccumulation potential in natural water bodies.^109^ The long-term ecological impacts of AOPs are further complicated by the lack of standardized protocols for evaluating their performance and by-products. Most studies focus on short-term laboratory-scale experiments, which may not accurately reflect real-world conditions. For example, the degradation efficiency of AOPs can vary significantly depending on factors such as pH, temperature, and the presence of organic matter, which are often not accounted for in laboratory studies. Moreover, the interaction of AOP-treated wastewater with other environmental stressors, such as climate change and eutrophication, remains poorly understood.^176^

Furthermore, the energy consumption and carbon footprint associated with AOPs raise questions about their sustainability.^177^ While AOPs are effective in removing antidepressants, their reliance on energy-intensive processes like UV irradiation or ozone generation may offset their environmental benefits. Life cycle assessments (LCAs) of AOPs are limited, and existing studies often overlook the long-term ecological trade-offs associated with these technologies.^178^

#### 7.3.2 Need for optimization and integration with existing treatment systems

AOPs, including photocatalysis, Fenton processes, ozonation, and UV-based systems, generate highly reactive species, such as hydroxyl radicals (˙OH), that can effectively degrade antidepressants into less harmful byproducts. Despite their efficacy, the standalone application of AOPs is often energy-intensive and costly, limiting their widespread adoption. Optimizing operational parameters, such as pH, catalyst dosage, oxidant concentration, and reaction time, is crucial for enhancing degradation efficiency and reducing operational costs.^116^ For instance, studies have shown that the degradation of fluoxetine, a typical SSRI, can be significantly improved by optimizing UV/H_2_O_2_ process conditions. Similarly, the use of heterogeneous catalysts in photocatalytic systems has been shown to enhance the removal efficiency of sertraline while minimizing energy consumption.^144^

Integrating AOPs with conventional biological treatment systems, such as activated sludge processes, offers a synergistic approach to improve overall treatment performance. Biological systems effectively remove bulk organic matter but often fail to eliminate trace organic contaminants, such as antidepressants.^179^ By coupling AOPs as a pre-treatment or post-treatment step, the biodegradability of wastewater can be enhanced, and the formation of toxic intermediates can be minimized. For example, pre-ozonation has improved the biodegradability of sewage containing citalopram, facilitating its subsequent removal in biological reactors. Conversely, post-treatment AOPs can be employed to polish effluent and ensure the complete degradation of residual antidepressants and their metabolites.^139^

The integration of AOPs with membrane filtration technologies, such as nanofiltration or reverse osmosis, also holds promise for the removal of antidepressants. Membrane processes can effectively concentrate pollutants, reducing the volume of wastewater requiring AOP treatment and lowering operational costs.^180^ Additionally, hybrid systems combining AOPs with adsorption processes using activated carbon or biochar have demonstrated enhanced removal efficiencies for antidepressants such as venlafaxine.^181^

Despite these advancements, challenges remain in scaling up and implementing AOP-based systems in real-world wastewater treatment plants. The variability in wastewater composition, the potential formation of toxic byproducts, and the high energy demands of AOPs necessitate further research and development.^10^ Pilot-scale studies and life cycle assessments are essential for evaluating the feasibility and environmental impacts of integrated systems. Moreover, developing cost-effective and sustainable catalysts, such as metal–organic frameworks (MOFs) or carbon-based materials, could further enhance the practicality of AOPs.^182^

## 8 Future perspectives

### 8.1 Innovative approaches

#### 8.1.1 Development of novel catalysts

AOPs, including photocatalysis, Fenton-based processes, and ozonation, generate highly reactive species, such as hydroxyl radicals (˙OH), to degrade organic pollutants. The performance of these processes is heavily dependent on the catalysts used. Recent research has focused on developing heterogeneous catalysts, which offer advantages such as reusability, stability, and ease of separation.^183^ For instance, titanium dioxide (TiO_2_)-based photocatalysts have been widely studied due to their high photocatalytic activity and chemical stability. However, the wide bandgap of TiO_2_ limits its efficiency under visible light. To address this, researchers have developed doped TiO_2_ catalysts, such as nitrogen-doped TiO_2_, which exhibit enhanced visible light absorption and improved degradation efficiency for antidepressants like fluoxetine and sertraline.^184^

Another promising approach is using metal–organic frameworks (MOFs) as catalysts in AOPs. MOFs possess high surface areas, tunable porosity, and active sites that can be functionalized for specific applications. For example, Fe-based MOFs have been employed as Fenton-like catalysts for the degradation of antidepressants, demonstrating high efficiency in generating ˙OH radicals. Additionally, incorporating noble metals, such as palladium or platinum, into MOFs has enhanced their catalytic activity and stability.^185^

Perovskite-based catalysts have also gained attention due to their unique electronic properties and high catalytic activity. LaCoO_3_ and related perovskites have been used in catalytic ozonation processes, achieving significant degradation of antidepressants like citalopram and venlafaxine. Furthermore, the development of carbon-based catalysts, such as graphene oxide and carbon nanotubes, has provided new opportunities for AOPs. These materials can act as catalysts and adsorbents, facilitating the removal of antidepressants through synergistic mechanisms.^186^

Despite these advancements, challenges remain in the practical application of novel catalysts. Issues such as catalyst deactivation, secondary pollution, and high production costs must be addressed. Future research should focus on developing cost-effective, environmentally friendly catalysts with long-term stability and high selectivity for the synthesis of antidepressants. Additionally, integrating AOPs with other treatment technologies, such as membrane filtration or biological processes, could enhance removal efficiency.^187^

#### 8.1.2 Hybrid systems combining AOPs with biological and physical methods

Biological treatments, such as activated sludge processes, biofilms, or constructed wetlands, are cost-effective and environmentally friendly; however, they often fail to completely degrade persistent pharmaceuticals, including antidepressants. Hybrid systems that integrate AOPs with biological treatments can overcome these limitations. For example, pre-treatment with AOPs can transform antidepressants into biodegradable intermediates, which are then efficiently mineralized by microorganisms.^188^ A study demonstrated that combining ozonation with a moving bed biofilm reactor significantly enhanced the removal of sertraline and fluoxetine. Similarly, a sequential photocatalytic-biological process achieved over 90% degradation of venlafaxine, highlighting the synergistic effects of such hybrid systems. Physical methods, such as adsorption and membrane filtration, effectively remove many contaminants but often encounter challenges, including adsorbent saturation or membrane fouling.^189^ Hybrid systems integrating AOPs with physical methods can address these issues. For instance, photocatalysis combined with membrane filtration can degrade antidepressants while reducing membrane fouling through the oxidative breakdown of organic foulants. Additionally, AOPs can regenerate adsorbents, such as activated carbon, extending their lifespan and improving cost-effectiveness. A study demonstrated the successful regeneration of activated carbon using Fenton oxidation, enabling its reuse to remove citalopram.^116^

### 8.2 Sustainability

#### 8.2.1 Use of renewable energy sources

The escalating presence of antidepressants in wastewater has emerged as a significant environmental concern due to their persistence, bioaccumulation potential, and adverse ecological effects. Conventional wastewater treatment methods often fail to effectively degrade these pharmaceuticals, necessitating the use of advanced treatment technologies.^190^ Advanced Oxidation Processes (AOPs) have gained prominence for their ability to generate highly reactive species, such as hydroxyl radicals (OH˙), to degrade recalcitrant organic pollutants like antidepressants.^191^ However, the high energy demands of AOPs, traditionally met by fossil fuel-based sources, pose sustainability challenges. Integrating renewable energy sources (RES) into AOPs offers a promising solution to enhance environmental sustainability while effectively removing antidepressants from wastewater. This review explores recent advancements in this domain, focusing on solar, wind, and bioenergy applications in AOPs.^105^

AOPs, including photocatalysis, Fenton-based processes, ozonation, and electrochemical oxidation, rely on energy-intensive mechanisms to produce reactive radicals. Antidepressants such as fluoxetine, sertraline, and venlafaxine, frequently detected in wastewater, resist biodegradation, making AOPs an ideal treatment choice.^167^ However, the operational costs and carbon footprint associated with ultraviolet (UV) lamps or electrical energy inputs have driven research toward renewable energy alternatives.^167^ Solar energy, in particular, has been widely investigated due to its abundance and compatibility with photocatalytic AOPs. Studies have demonstrated that solar-driven photocatalysis using TiO_2_ catalysts effectively degrades antidepressants under natural sunlight, resulting in significant reductions in energy costs.^192^ The Fenton process, another potent AOP, has also been adapted to harness solar energy. Photo-Fenton systems, which combine Fe^2+^, H_2_O_2_, and solar irradiation, enhance radical generation while minimizing energy consumption. Researchers successfully applied solar photo-Fenton technology to remove venlafaxine from wastewater, achieving near-complete degradation within a few hours. This approach leverages solar photons as a renewable driver, reducing reliance on artificial UV sources. Similarly, sulfate radical-based advanced oxidation processes (AOPs), such as persulfate activation, have been coupled with solar energy, demonstrating high efficiency in the degradation of antidepressants.^165^

Electrochemical Advanced Oxidation Processes (EAOPs) offer another avenue for Renewable Energy System (RES) integration, particularly with wind or solar-powered systems. Although less explored, wind energy has the potential to power EAOPs in regions with consistent wind availability. Bioenergy, derived from microbial fuel cells (MFCs), has also emerged as a novel RES for AOPs. MFCs are coupled with electro-Fenton processes, achieving sustainable removal of antidepressants while generating bioelectricity.^193^ Despite these advancements, challenges remain, including the intermittency of solar and wind energy, high initial costs, and the need for efficient energy storage systems. Hybrid systems combining AOPs with membranes or biological treatments powered by RES show promise for overcoming these limitations.^194^ Future research should optimize catalyst design, integrate multiple RES, and conduct life-cycle assessments to ensure economic and environmental viability.^104^

#### 8.2.2 Green chemistry principles in AOP design

Integrating green chemistry principles into AOP design enhances sustainability, aligning with the global effort to develop environmentally friendly wastewater treatment solutions. This review examines how these principles can optimize AOPs for the removal of antidepressants, with a focus on efficiency, minimal waste, and eco-friendly methodologies. Green chemistry emphasizes the design of processes that reduce or eliminate hazardous substances, maximize atom economy, and utilize renewable resources.^195^ In AOPs, this translates to selecting catalysts, oxidants, and reaction conditions that minimize environmental footprints. For instance, photocatalysis using titanium dioxide (TiO_2_) under solar irradiation exemplifies a green approach that harnesses renewable energy and reduces reliance on energy-intensive UV lamps. Studies have demonstrated its efficacy in degrading antidepressants like fluoxetine and sertraline, achieving over 90% removal under optimized conditions.^196^

Another green chemistry principle is the use of safer chemicals and solvents. Traditional AOPs, such as the Fenton process (Fe^2+^/H_2_O_2_), often require acidic conditions (pH ∼3), generating iron sludge as a byproduct. To address this, heterogeneous Fenton-like systems using iron-based catalysts (*e.g.*, Fe_3_O_4_ nanoparticles) have been developed, enabling operation at neutral pH and reducing secondary waste.^197^ Research has shown these systems effectively degrade venlafaxine with minimal sludge formation. Ozonation, another AOP, aligns with green chemistry by avoiding chemical residues combined with catalysts like activated carbon, enhancing ˙OH production while minimizing ozone overuse. Studies report near-complete removal of citalopram within 30 minutes using catalytic ozonation, highlighting its efficiency and reduced ecological impact.^198^

Energy efficiency is a cornerstone of green chemistry. Combining AOPs, such as UV/H_2_O_2_ with ultrasound (sonolysis), reduces energy demands by synergistically generating radicals. This hybrid approach has successfully degraded trazodone, cutting treatment time by 40% compared to standalone processes. Similarly, electrochemical AOPs using boron-doped diamond electrodes offer high oxidation power with low energy consumption, effectively mineralizing paroxetine.^173^ Preventing waste is critical in green AOP design. Biochar-supported photocatalysts derived from agricultural waste exemplify this principle by repurposing biomass while degrading antidepressants, such as duloxetine, with high efficiency. These systems also reduce the need for synthetic catalysts, lowering costs and environmental impact.^180^ Despite these advances, challenges remain, including scalability and byproduct toxicity. Integrating green chemistry requires striking a balance between efficiency and sustainability, often necessitating the use of renewable energy sources and biodegradable catalysts. Future research should focus on life cycle assessments to quantify environmental benefits and explore natural photocatalysts, such as zinc oxide, under solar light.^199^

### 8.3 Policy and regulation

#### 8.3.1 Need for stricter regulations on pharmaceutical discharge

Current regulations in many regions, such as the U.S. Clean Water Act and the EU Water Framework Directive, set broad limits on organic pollutants but often lack specific thresholds for pharmaceuticals. This regulatory gap allows untreated or partially treated antidepressant residues from households, hospitals, and pharmaceutical manufacturing facilities to enter municipal wastewater treatment plants (WWTPs), which are often inadequately equipped to remove them.^200^ Studies estimate that up to 70% of antidepressants pass through conventional treatment unchanged, necessitating AOPs as a tertiary solution. However, without curbing initial discharge, AOPs become a costly Band-Aid rather than a sustainable fix.^201^ Stricter regulations could mandate pre-treatment at pharmaceutical production facilities, where effluents often contain antidepressant concentrations that are orders of magnitude higher than domestic wastewater. Research shows that implementing source control, such as adsorption or membrane filtration, reduces the pollutant load entering WWTPs, enhancing AOP efficiency downstream. For instance, Fenton-based AOPs achieve over 95% removal of citalopram when inlet concentrations are moderated, but efficiency drops significantly under high loads.^202^

The environmental fate of antidepressant metabolites and AOP byproducts further justifies regulatory reform. While AOPs like photocatalysis with TiO_2_ or ozonation degrade parent compounds effectively, incomplete mineralization can produce transformation products with unknown toxicity. Stricter discharge limits could enforce monitoring of these byproducts, pushing industries to adopt greener AOP designs or alternative disposal methods, such as incineration of pharmaceutical waste.^203^ The economic and logistical challenges of AOPs also underscore the need for upstream regulation. Processes like UV/H_2_O_2_ or electrochemical oxidation, while effective against paroxetine and duloxetine, require significant energy and chemical inputs, making them unsustainable for large-scale use without reduced influent loads. Studies suggest that regulatory caps on discharge could decrease AOP operational costs by 30–50%, redirecting resources to optimize treatment technologies. Global precedents, such as Switzerland's micropollutant legislation, demonstrate that stricter standards work.^204^ By mandating WWTP upgrades and industrial accountability, Switzerland reduced effluent pharmaceutical residues by over 80%. Similar policies could incentivize pharmaceutical companies to adopt cleaner production practices, easing the burden on AOPs.^205^

#### 8.3.2 Incentives for adopting advanced treatment technologies

Adopting advanced treatment technologies in water and wastewater management is critical for addressing water scarcity, pollution, and stringent regulatory requirements. These technologies, including membrane filtration, advanced oxidation processes (AOPs), and nutrient recovery systems, offer significant benefits in terms of efficiency, sustainability, and environmental protection. However, their implementation often faces barriers, including high capital costs, operational complexity, and limited awareness. To overcome these challenges, various incentives are essential to encourage the adoption of advanced treatment technologies.^202^

##### 8.3.2.1 Economic incentives

Economic benefits are a primary driver for adopting AOPs. While initial setup costs for technologies like photocatalysis (*e.g.*, TiO_2_/UV) or electrochemical oxidation (*e.g.*, boron-doped diamond electrodes) are high, long-term savings can offset these expenses. For instance, solar-driven photocatalysis reduces energy costs by leveraging renewable sunlight, achieving over 85% removal of fluoxetine in pilot studies. Additionally, hybrid AOPs, such as UV/H_2_O_2_ combined with ozonation, enhance efficiency, reducing treatment time and operational costs.^193^ Governments and institutions can incentivize adoption by offering subsidies, tax breaks, or low-interest loans to WWTPs that invest in these systems. Research highlights that such financial support has spurred the implementation of AOP in regions like Europe, where cost recovery is achieved within 5 to 7 years.

##### 8.3.2.2 Environmental incentives

The environmental benefits of AOPs provide compelling incentives. Unlike conventional treatments (*e.g.*, activated sludge), AOPs effectively degrade antidepressants like sertraline and venlafaxine, preventing their accumulation in aquatic ecosystems. Studies show that ozonation can achieve near-complete removal of citalopram within 20 minutes, minimizing ecotoxicological risks. Moreover, integrating green chemistry principles—such as using biodegradable catalysts like biochar or minimizing chemical inputs—reduces secondary pollution, aligning with sustainability goals. These environmental gains incentivize WWTPs to adopt AOPs, enabling them to meet stringent discharge standards and protect biodiversity, particularly in sensitive watersheds.^181^

##### 8.3.2.3 Regulatory incentives

Regulatory frameworks play a pivotal role in adoption. Many countries lack specific limits for pharmaceuticals in wastewater; however, emerging policies, such as the European Union's Water Framework Directive, are tightening regulations for micropollutants. Compliance with these standards often requires advanced treatment methods, such as Advanced Oxidation Processes (AOPs). For example, Switzerland has mandated tertiary pharmaceutical treatment, which has led to the increased use of ozonation and Fenton processes. Incentives such as relaxed permitting processes, certification programs, or funding for pilot projects can encourage WWTPs to transition to AOPs, ensuring regulatory compliance while addressing antidepressant pollution.^173^

##### 8.3.2.4 Social and public health incentives

Public awareness of the health implications of pharmaceutical pollution—such as antidepressant residues in drinking water—creates social pressure for more advanced treatments. AOPs mitigate these risks by ensuring safer water supplies and fostering community support for their adoption. Research demonstrates that electrochemical AOPs can degrade paroxetine to non-toxic levels, enhancing public trust in water quality. Incentives like public-private partnerships or educational campaigns can amplify this momentum, encouraging investment in AOP infrastructure and aligning with corporate social responsibility goals for utilities.^116^

##### 8.3.2.5 Technological advancements as incentives

Innovations in AOP design lower barriers to adoption. Heterogeneous Fenton-like systems, using Fe_3_O_4_ nanoparticles, operate at neutral pH, reducing sludge disposal costs compared to traditional Fenton processes. Similarly, sono photocatalysis combines ultrasound and UV/H_2_O_2_ to degrade trazodone with 30% less energy than standalone methods. These advancements make AOPs more practical and cost-effective as intrinsic incentives for WWTPs to upgrade. Collaborative research and technology transfer programs can further incentivize adoption by providing access to cutting-edge solutions.

## 9 Conclusion

The increasing detection of antidepressants in aquatic environments highlights the urgent need for effective remediation strategies that extend beyond conventional wastewater treatment approaches. Antidepressants persist at trace concentrations and pose significant risks to aquatic life, altering behavior, bioaccumulating in tissues, and disrupting ecosystem dynamics. While conventional treatment methods, such as adsorption and biodegradation, offer partial removal, they often fail to achieve complete mineralization, necessitating the use of more advanced technologies.

Advanced Oxidation Processes (AOPs) have demonstrated superior efficiency in breaking down antidepressants into non-toxic byproducts by generating highly reactive radicals. Among these, photocatalysis, Fenton-like reactions, ozonation, and sulfate radical-based oxidation have shown promising results in removing persistent pharmaceutical contaminants. However, challenges such as operational costs, byproduct toxicity, and energy consumption must be addressed to enhance the feasibility of AOPs for large-scale implementation. Future research should focus on optimizing AOPs by integrating renewable energy sources, developing selective catalysts, and minimizing secondary pollution. Combining AOPs with existing treatment methods presents a viable strategy for achieving sustainable and efficient removal of antidepressants from wastewater, ultimately reducing their environmental impact and safeguarding aquatic ecosystems.

## Conflicts of interest

The author declares that there are no conflicts of interest regarding the publication of this paper.

## Abbreviations

WWTPs: Waste water treatment plants
STPs: Sewage treatment plants
SSRIs: Selective serotonin reuptake inhibitors
SNRIs: Serotonin-norepinephrine reuptake inhibitors
AOPs: Advanced oxidation processes
ATDs: Antidepressants
TCAs: Tri cyclic antidepressants
NDRI: Norepinephrine-dopamine reuptake inhibitor
GAD: Generalized anxiety disorder
PTSD: Post-traumatic stress disorder
ATD: Advanced treatment discharge
OCD: Obsessive-compulsive disorder
MDD: Major depressive disorder
PTSD: Post-traumatic stress disorder
MAOIs: Monoamine oxidase inhibitors
NARI: Noradrenaline reuptake inhibitor
NaSSA: Noradrenergic and specific serotonergic antidepressant
EAOPs: Electrochemical advanced oxidation processes
RES: Renewable energy system
ROS: Reactive oxygen species
MFCs: Microbial fuel cells
MOFs: Metal–organic frameworks

## Data Availability

This work does not include any available data, as it is a review article.
